# Identification of tryptophan metabolism-related genes in immunity and immunotherapy in Alzheimer’s disease

**DOI:** 10.18632/aging.205220

**Published:** 2023-11-20

**Authors:** Zhenyan Song, Zixuan Wu, Rongsiqing Luo, Chunxiang He, Ze Li, Miao Yang, Wenjing Yu, Jiawei He, Sisi Deng, Shaowu Cheng

**Affiliations:** 1 School of Integrated Chinese and Western Medicine, Hunan University of Chinese Medicine, Changsha 410208, Hunan, China; 2 Key Laboratory of Hunan Province for Integrated Traditional Chinese and Western Medicine on Prevention and Treatment of Cardio-Cerebral Diseases, Hunan University of Chinese Medicine, Changsha 410208, Hunan, China

**Keywords:** Alzheimer’s disease, tryptophan metabolism-related genes, DEGs, WGCNA, bioinformatics

## Abstract

Recent studies have highlighted the significant involvement of tryptophan metabolism in the pathogenesis of Alzheimer’s disease (AD). However, a comprehensive investigation of the precise role of tryptophan metabolism in the context of AD is still lacking. This study employed a bioinformatics approach to identify and validate potential tryptophan metabolism-related genes (TrpMgs) associated with AD. The discovery of TrpMgs was facilitated through the intersection of the Weighted Gene Co-expression Network Analysis (WGCNA) test and 17 known tryptophan metabolism pathways. Subsequently, the putative biological functions and pathways of the TrpMgs were elucidated using Gene Set Variation Analysis (GSVA). Furthermore, the Least Absolute Shrinkage and Selection Operator (LASSO) method was applied to identify hub genes and evaluate the diagnostic efficiency of the 5 TrpMgs in distinguishing AD. The relationship between hub TrpMgs and clinical characteristics was also investigated. Finally, *in vivo* verification of the five TrpMgs was performed using APP/PS1 mice. We identified 5 TrpMgs associated with AD, including propionyl-CoA carboxylase subunit beta (PCCB), TEA Domain Transcription Factor 1 (TEAD1), Phenylalanyl-TRNA Synthetase Subunit Beta (FARSB), Neurofascin (NFASC), and Ezrin (EZR). Among these genes, PCCB, FARSB, NFASC, and TEAD1 showed correlations with age. In the hippocampus of APP/PS1 mice, we observed down-regulation of FARSB, PCCB, and NFASC mRNA expressions. Furthermore, PCCB and NFASC protein expressions were also down-regulated in the cerebral cortex and hippocampus of APP/PS1 mice. Our study paves the way for future research aimed at unraveling the intricate mechanisms underlying tryptophan metabolism dysregulation in AD and its therapeutic implications.

## INTRODUCTION

Alzheimer’s disease (AD) is a neurodegenerative disorder characterized by a progressive and insidious onset. It presents clinically with various manifestations including memory impairment, aphasia, apraxia, agnosia, visuospatial skills impairment, executive dysfunction, and changes in personality and behavior [1]. Currently, approximately 6.5 million Americans aged 65 and older are affected by AD, and this number is projected to increase to 13.8 million by 2060 [2]. In China alone, there are approximately 10 million AD patients, and the annual cost of AD treatment is expected to reach $1.8 trillion by 2050 [3]. Aging is the greatest risk factor for AD, with the incidence of the disease sharply increasing with age: 5.0% in individuals aged 65 to 74, 13.1% in those aged 75 to 84, and 33.2% in individuals aged 85 and older [2]. One of the major challenges in treating AD lies in the incomplete understanding of its pathogenesis. The characteristic histopathological features of AD include the presence of abnormal fibrillary deposits known as amyloid β (Aβ) plaques and neurofibrillary tangles composed of Tau protein. It is widely accepted that the imbalanced generation and clearance of Aβ play a crucial role in neuronal degeneration and the development of dementia. The accumulation of Aβ plaques between neurons in the brain exerts neurotoxic effects, ultimately leading to neuronal degeneration [4].

In the last two decades, the progress of drug development for AD has faced significant challenges. While a few drugs such as Memantine and Donepezil have been successfully marketed, the majority of AD clinical trials have failed to demonstrate efficacy [5]. Nevertheless, despite these setbacks, the scientific community remains optimistic about the development of AD therapeutics and continues to explore novel research strategies. In recent studies, AD has been increasingly recognized as an autoimmune disorder, wherein Aβ acts as an immune peptide and initiates a series of events in the innate immune system. This process triggers a chronic, self-perpetuating, progressive autoimmune cycle characterized by microglial activation, release of pro-inflammatory cytokines, tau protein aggregation, and synaptic toxicity [6]. Besides, emerging evidence suggests that amino acid metabolic pathways may play a crucial role. Of particular interest is the role of tryptophan metabolism, as it has been implicated in the molecular pathogenesis of several neurological diseases [7]. Researchers are actively investigating the potential involvement of tryptophan metabolism in AD, recognizing its significance as a potential therapeutic target.

Tryptophan, an indispensable amino acid in mammals, cannot be synthesized endogenously and must be obtained through dietary sources. Its primary role lies in protein synthesis, but it is also metabolized into various bioactive compounds through two distinct pathways: the serotonin pathway, leading to melatonin production, and the kynurenine pathway, resulting in the formation of niacin derivatives. These pathways generate biologically significant intermediary molecules that contribute to the regulation of neural function, immune response, and metabolism [8]. Moreover, Tryptophan metabolites play a role as endogenous compounds that modulate the immune response in AD. Specifically, indole-based and anthranilate-based metabolites have been identified as interacting directly with Aβ, exhibiting the ability to inhibit the oligomerization and aggregation of Aβ [9].

**Figure 1:**
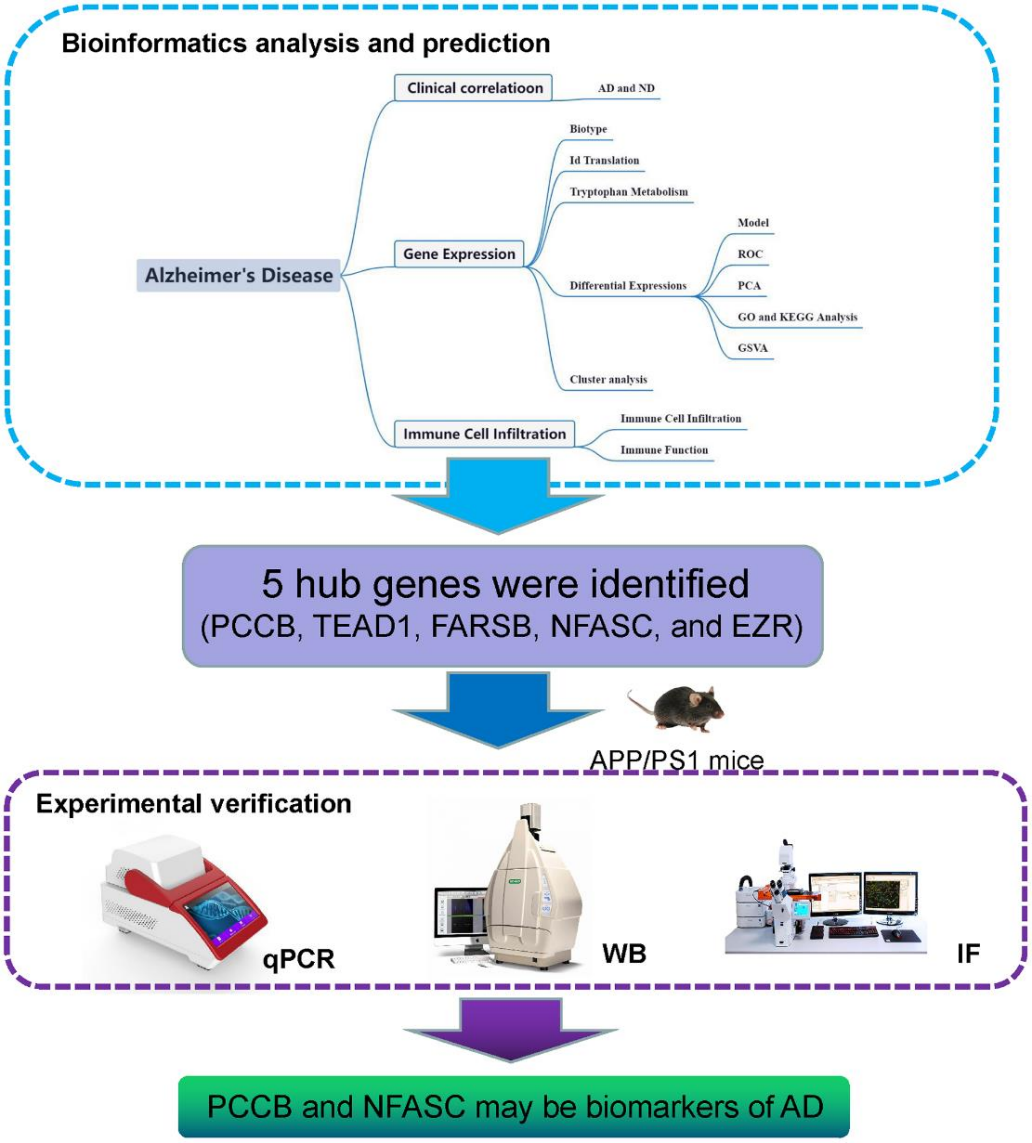
**Flow diagram.** The study utilized a systematic framework to investigate the role of tryptophan metabolism-related genes in Alzheimer’s disease (AD).

The modulation of tryptophan metabolism holds great potential in combination with immunotherapy for the treatment of AD, as it plays a crucial role in the immune and inflammatory response within the central nervous system. Targeting tryptophan metabolism, alongside immunotherapy, shows promise in the management of AD. In biomedical research, gene expression analysis has proven valuable in establishing associations between genes, diseases, and drugs. Researchers in the field of drug development have utilized this approach to identify changes in transcription and related molecular pathways associated with AD. For instance, some studies have explored the molecular pathways involved in AD pathogenesis using gene expression data obtained from resources like the Gene Expression Omnibus (GEO), focusing on age-related genes and ferroptosis-related genes [10, 11]. This approach offers valuable insights into the pathophysiological processes underlying AD and aids in the identification of potential drug targets from multiple perspectives. However, to date, no studies have investigated the role of TrpMgs in the development of AD. Therefore, our work aims to provide a comprehensive evaluation of the immunotherapeutic potential of TrpMgs in the context of AD (Figure 1).

## MATERIALS AND METHODS

### Raw data processing

GEO database was utilized using the following search criteria: Series: GSE5281, GSE37263, GSE106241, GSE132903, and GSE63060; Platform: GPL570, GPL5175, GPL6947, GPL24170, and GPL10558. The datasets GSE5281, GSE37263, GSE106241, and GSE132903 were utilized as training and test groups, while GSE63060 served as the independent test group. The search strategy employed was as follows: (‘Alzheimer’s disease’ [MeSH] mRNA [All Fields] and normal) AND (‘Homo sapiens’ [Organism] AND ‘Non-coding RNA profiling by array’ [Filter]). To identify relevant tryptophan metabolism-related genes, the Molecular Signatures Database (MSigDB) was employed, resulting in the retrieval of 40 genes (Appendix 1).

### Analysis of DEGs

Transcription data obtained from Perl (https://github.com/Perl) were processed to obtain precise mRNA data. The obtained IDs were converted into corresponding gene names. Following this, data standardization was performed on GSE5281, GSE37263, GSE106241, and GSE132903 using the “normalize Between Arrays” function from the “limma” package. Principal Component Analysis (PCA) was conducted using the “factoextra” package to examine the variability in the data. To identify Differentially Expressed Genes (DEGs) between AD and non-demented controls (ND), statistical analysis was performed. The DEGs were selected based on the criteria of |Fold Change (FC)| > 1 and p-value < 0.05. To visually represent the significantly deregulated genes, a heatmap was generated using the “ggplot2” and “ComplexHeatmap” packages.

### Immune cell infiltration

The immune cell components in adipose tissue were analyzed via CIBERSORT. We built barplot and corplot with the limma package to show the results of immune cells.

### Cluster analysis

Cluster analysis was performed using the limma and ConsensusClusterPlus packages. By setting the clustering variable (k) to 2, we observed a strong correlation within each cluster and a weak correlation between clusters. The tryptophan metabolism-related genes associated with prognosis were classified into cluster 1 and cluster 2 based on this analysis. Additionally, a consensus score was calculated to further evaluate the results [12]. Furthermore, the limma package was employed to identify specific gene changes between subtypes and tissue types, allowing for the detection of differential gene expression patterns [13].

### Enrichment analysis

To investigate the biological functions and pathways associated with the DEGs, we utilized Gene Ontology (GO) and the Kyoto Encyclopedia of Genes and Genomes (KEGG). The DEGs were analyzed to determine their involvement in various biological processes, molecular functions, and cellular components using R. Specifically, we obtained the “c5.go.bp.v7.5.1.symbols” gene sets from MSigDB to perform the analysis.

To quantify the process scores, we employed the GSVA (Gene Set Variation Analysis) method, which is implemented in the “GSVA” package. This allowed us to compute the scores associated with different biological processes regulated by the DEGs. By utilizing this approach, we gained insights into the functional implications of the tryptophan metabolism-related genes in AD.

### Co-expression gene identification

To explore the genetic processes involved in the development of AD, we employed the WGCNA algorithm. This algorithm allows for the clustering of genes into distinct modules and the examination of correlations between these modules and disease features. We utilized the “WGCNA” package to construct a co-expression network. To ensure robust results, we focused on genes with the highest 25% variance from the GSE132903 dataset. The dynamic cutting tree approach with a threshold of 0.25 was employed to merge modules. Several criteria were employed during the construction of the co-expression network. Firstly, the soft threshold power (β) was determined based on the scale-free topology requirement, aiming for a high independence value (R^2 = 0.85). The selection of the soft threshold was carried out using the select Soft Threshold function. Additionally, a minimum of 30 genes was set as the threshold for each module.

To identify potential relationships between the modules and clinical variables of the patients, Pearson correlation analysis was performed. This analysis allowed us to assess the degree of correlation between module expression patterns and clinical characteristics, providing valuable insights into the underlying mechanisms of AD.

### Tryptophan metabolism-related genes identification

To identify the tryptophan metabolism-related genes, we performed an intersection analysis of the DEGs derived from major modules obtained through WGCNA, Gln, and cluster hubGenes. Visualization of the overlapping genes was achieved using Vnnmap. Furthermore, we investigated the biological processes and enrichment pathways associated with these genes. Separately, the datasets GSE5281, GSE37263, GSE106241, and GSE132903 were divided into training cohorts after identifying the hub tryptophan metabolism-related genes. To identify the hub DEGs, we utilized the “glmnet” package and selected the smallest lambda value as the optimal parameter. Subsequently, we calculated the DEGs’ predictive scores in each sample. To assess the diagnostic and discriminative potential of the tryptophan metabolism-related genes in AD and ND individuals, we conducted receiver operating characteristic curve analysis. The GSE63060 dataset was used for external validation.

Finally, we performed prognosis calculations on the test group by matching samples based on age-related clinical information. We also explored the correlation between these genes and age, providing valuable insights into their relationship with the aging process.

### Drug-gene interactions

With the advancement of bioinformatics, the construction of biological models and the identification of efficient biomarkers has become more significant in the diagnosis and prevention of clinical disorders. Even if the biomarkers are established, the crucial issue is determining how to use them in the clinic. As a result, medication prediction based on successful indicators will be critical in the future prevention and treatment of AD. Validated biomarkers provide some reference for clinical treatment. Therefore effective drug prediction is very important. We used the DGIdb database (https://dgidb.genome.wustl.edu/) to make drug predictions for both the obtained hub genes and the intersection gene in the eXtreme Gradient Boosting (XGB) model.

### Animals

Male APP/PS1 double transgenic mice (B6C3-Tg (APPswe, PSEN1dE9) 85Dbo/J) at the age of 12 months were obtained from Nanjing Junke Biotechnology Corporation, Ltd. (Nanjing, China). The experimental animal production license number was SCXK (SU) 2017-0003. The control group consisted of male C57BL/6J mice, also 12 months old, obtained from Hunan SJA Laboratory Animal Co., Ltd. (Changsha, China). The experimental animal production license number was SCXK (Xiang) 2019-0004. The mice were housed in a specific pathogen-free (SPF) animal room at Hunan University of Chinese Medicine. The animal reproduction and genotypic identification followed previously published methods [14].

### Quantitative real time PCR (qPCR)

Rat hippocampal tissues were used for total RNA extraction using TRlzol (15596-026, Thermo Scientific, Shanghai, China). The extracted RNA was then subjected to reverse transcription using a reverse transcription cDNA kit (RR047A, Takara, Dalian, China) according to the provided instructions, resulting in cDNA synthesis. Two-step amplification of the target genes was conducted using SYBR Green (RR820L, Takara, Dalian, China) on a Real-time PCR instrument (T100, Bio-Rad, USA).

The amplification reaction procedure consisted of an initial denaturation step at 95° C for 5 minutes, followed by 40 cycles of denaturation at 95° C for 30 seconds and annealing/extension at 58° C for 30 seconds. Ct values, representing the cycle threshold, were recorded for each gene, and subsequent statistical analysis of the data was performed using the 2^-ΔΔCt^ method. The primer sequences used in the PCR amplification are presented in Table 1.

**Table 1:** Primer sequences of PCR.

**Name**	**Forward sequence (5’-3’)**	**Reverse sequence (5’-3’)**	**Length (bp)**
PCCB	TGGCTTCGCAAGAATGAATGG	CCTTCCTGGTGATGACTGTGA	103
TEAD1	AACCGCTCGCCAATGTGT	GTGCTCCGTGTTCGCTATTC	190
FARSB	TGCTATTGGAACTCACGACTTG	GACAGGACCACACCATTGC	225
NFASC	ACACCAATAACCAGGCAGACA	ATGAAGCAGACGATGAGAAGGA	100
EZR	CACAGAGGCAGAGAAGAATGAG	TCAATGCGTTGCTTGGTGTT	189
β-actin	AGACCTCTATGCCAACACAGT	TCCTGCTTGCTGATCCACAT	210

### Western blotting

The hippocampus of mice was used for total protein extraction using RIPA lysate (cat. P0013B, Beyotime Biotechnology Co., Ltd., Shanghai, China) following the provided instructions. The protein concentration was determined using the BCA (23227, Thermo Scientific, Shanghai, China) method. Subsequently, the protein samples were loaded onto SDS-PAGE gels and subjected to electrophoretic separation. The separated proteins were then transferred to PVDF membranes (0.45 μm pore size, IPVH00010, Millipore Sigma Inc., Billerica, USA). To prevent non-specific binding, the membranes were blocked using 5% skim milk. The primary antibody (diluted at 1:1000) was incubated with the membranes overnight at 4° C, followed by incubation with the secondary antibody (diluted at 1:5000) at room temperature for 2 hours. Chemiluminescent signals were generated using an ECL chemiluminescence kit (32132, Thermo Scientific, Shanghai, China), and the blots were visualized using an imaging system (ChemiDoc™ XRS+, Bio-Rad, USA). The primary antibodies used in this study included Anti-PCCB Rabbit pAb (GB113237, Servicebio, Wuhan, China), Anti-Neurofascin Rabbit pAb (GB111351, Servicebio, Wuhan, China), and Anti-GAPDH mouse pAb (GB15002, Servicebio, Wuhan, China).

### Immunofluorescence

Mouse brain tissue samples were fixed using 4% paraformaldehyde, followed by paraffin embedding. The paraffin-embedded tissue blocks were then sectioned into 4 μm thick slices using a paraffin microtome (HM355S, Thermo Scientific, Shanghai, China). To prepare the tissue for immunostaining, the sections underwent steps including deparaffinization, antigen retrieval, blocking, and incubation with primary and secondary antibodies, as described in previous studies [15]. Specifically, cell transparency techniques were employed, and antigen retrieval was performed to enhance the antigenicity of the tissue. After blocking to reduce non-specific binding, the sections were incubated with primary antibodies, including PCCB antibody at a dilution of 1:200 and Neurofascin antibody at a dilution of 1:200. Following the primary antibody incubation, the sections were incubated with appropriate secondary antibodies. Restaining and tablet sealing procedures were carried out according to established protocols. Full image scanning and subsequent statistical analysis were performed using the TissueFAXS imaging system (Tissue Gnostics GmbH, Austria), allowing for comprehensive evaluation of the stained tissue sections.

### Statistical analysis

Statistical analysis was conducted using GraphPad Prism 8.0 Software. The data were expressed as mean ± standard deviation (SD). A two-group comparison was performed using the t-test. P-values less than 0.05 were considered statistically significant. For the identification of differentially expressed mRNAs, a threshold of FC greater than or equal to 2 and a p-value less than 0.05 were used for screening.

### Data availability statement

The datasets generated during and/or analyzed during the current study are available in the Appendix.

## RESULTS

### DEG identification and principal component analysis

**Figure 2:**
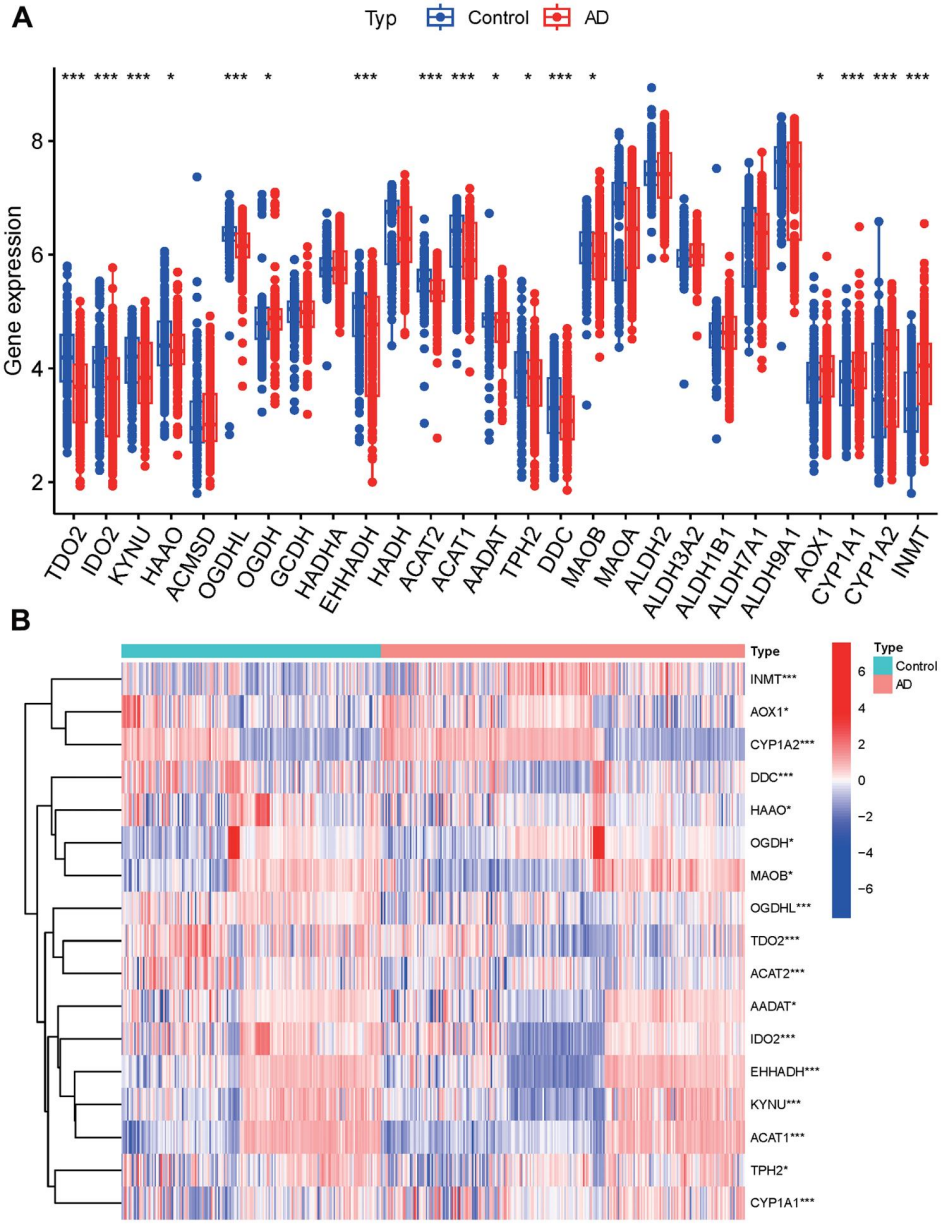
**Principal component analysis.** (**A**) Tryptophan metabolism-related genes. (**B**) Expression of tryptophan metabolism-related genes in clusters.

Among the 17 tryptophan metabolism-related genes examined, all showed significant differences between groups, except for ACMSD, GCDH, HADHA, HADH, MAOA, ALDH2, ALDH3A2, ALDH1B1, ALDH7A1, and ALDH9A1 (Figure 2A). These genes exhibited varying patterns of expression, with some genes clustering in the AD group and others in the control group. Specifically, the AD group showed increased expression of INMT, OGDH, MAOB, AADAT, IDO2, EHHADH, KYNU, ACAT1, TPH2, and CYP1A1. On the other hand, the control group exhibited increased expression of AOX1, CYP1A2, DDC, HAAO, OGDHL, TDO2, and ACAT2 (Figure 2B and Appendix 2).

### Expression of tryptophan metabolism-related genes

**Figure 3:**
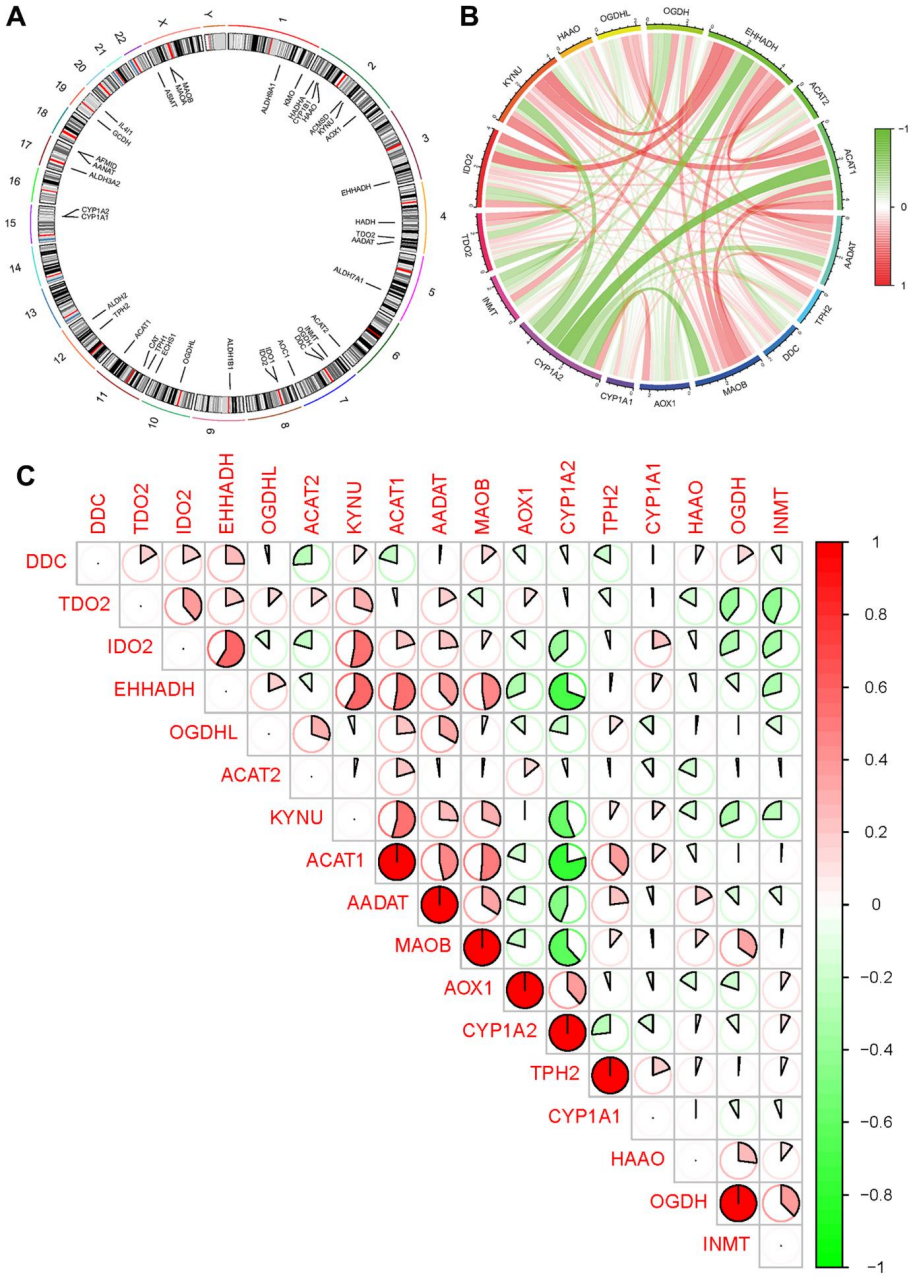
**Expression of tryptophan metabolism-related genes (TrpMgs).** (**A**) Expression of TrpMgs on sequences. (**B**, **C**) The correlation between TrpMgs and related genes.

To determine the chromosomal positions of the tryptophan metabolism-related genes, we performed calculations and visualized the data in a circular format (Figure 3A and Appendix 3). Additionally, in order to gain insights into the expression patterns of these genes, we conducted correlation analysis among them (Figure 3B, 3C).

### Immune cells

**Figure 4:**
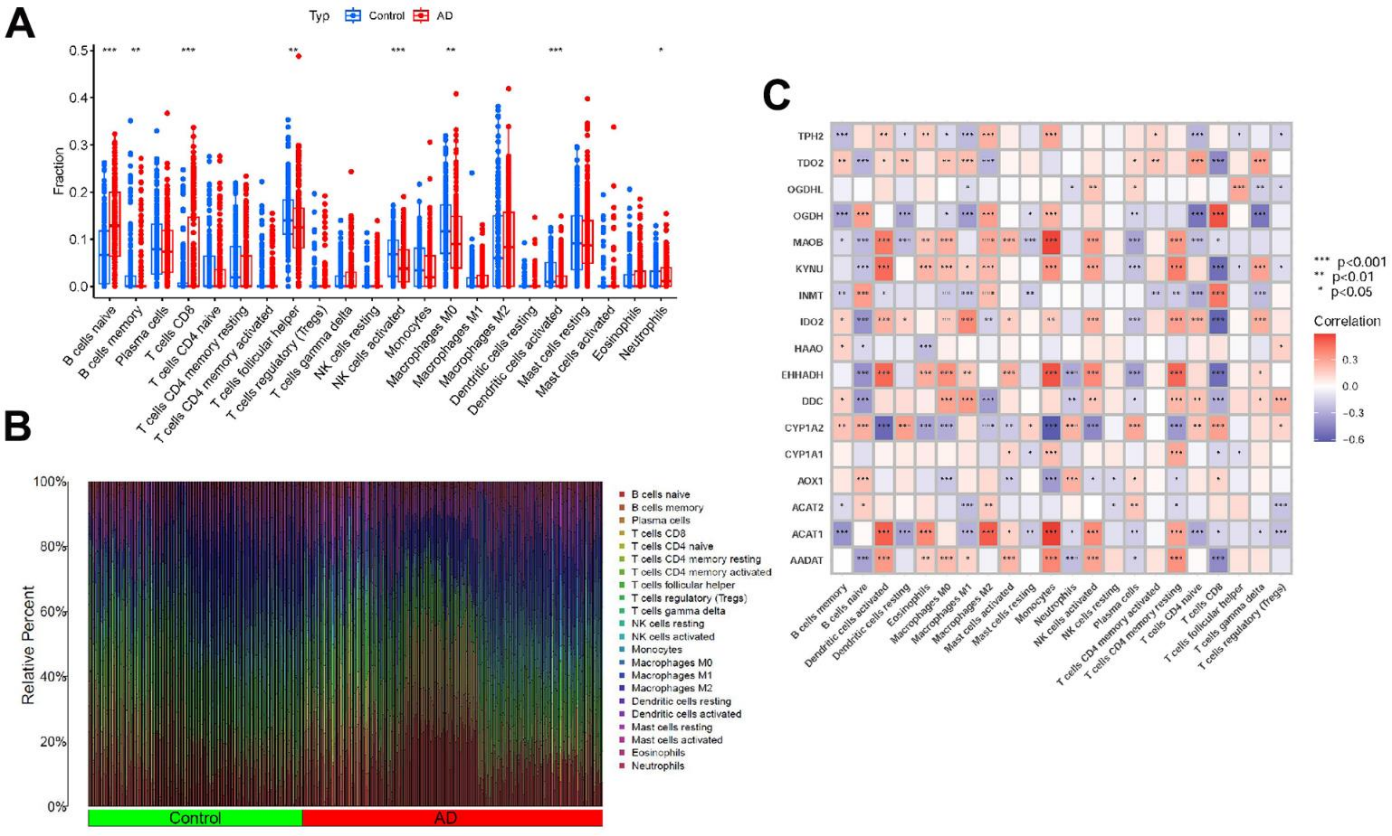
**Expression of immune cells.** (**A**, **B**) Expression of immune cells in different clusters. (**C**) Correlation between TrpMgs and immune cells.

The immune environment plays a critical role in the initiation and advancement of AD. To assess the composition of immune cell populations in adipose tissue, we utilized CIBERSORT. Subsequently, we visualized the results using bar plots and correlation plots to depict the immune cell components (Figure 4A, 4B). Furthermore, to elucidate the relationship between the expression of the tryptophan metabolism-related genes and immune cells, we conducted correlation analysis (Figure 4C).

### Cluster analysis

**Figure 5:**
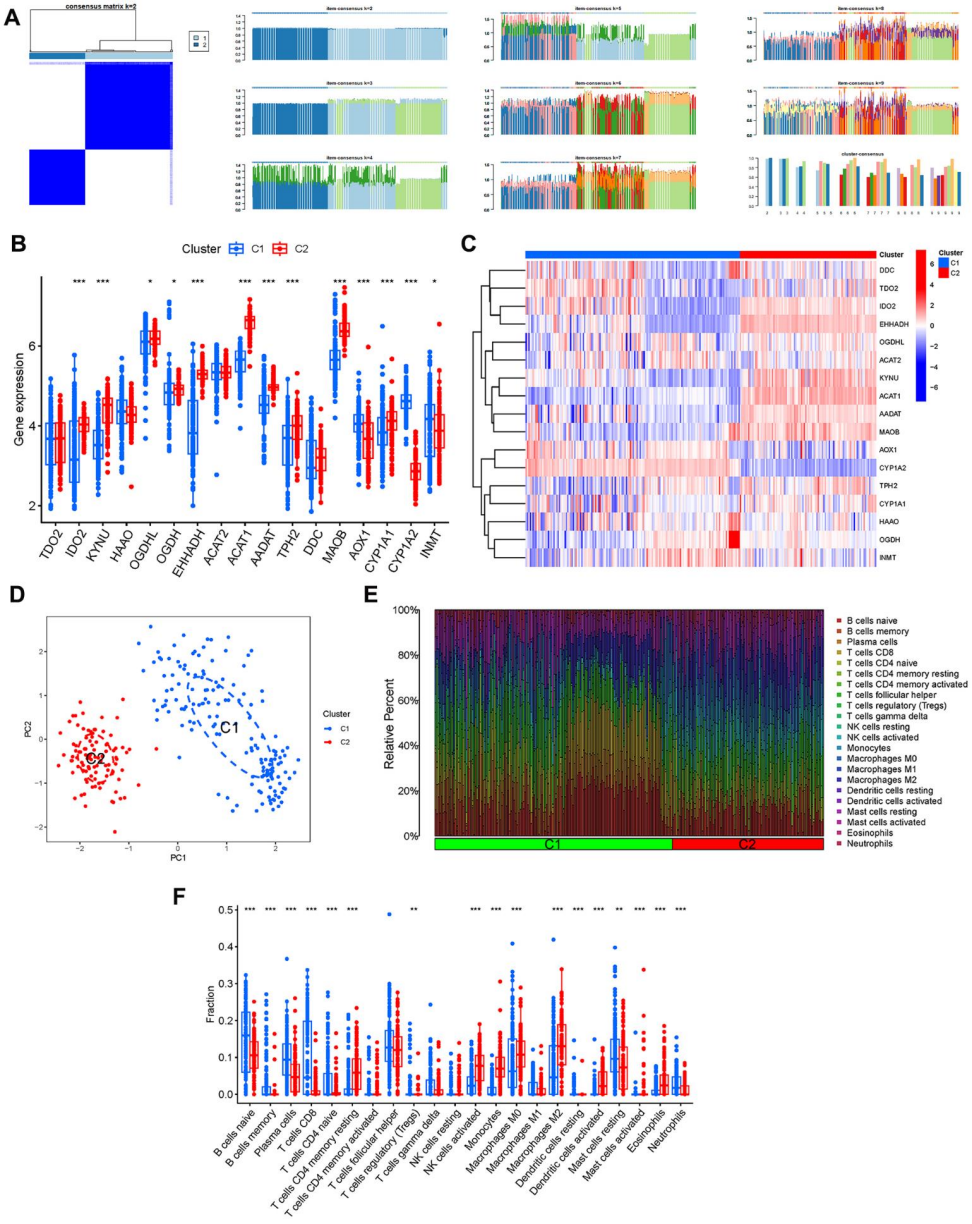
**Cluster analysis.** (**A**) Consensus clustering matrix. (**B**, **C**) Expression of the TrpMgs in different clusters. (**D**) PCA. (**E**, **F**) Immune cell infiltration of different clusters.

By setting the clustering variable (k) to 2, we observed that the intragroup correlations were the strongest and the intergroup correlations were the smallest, suggesting that patients with AD can be classified into two distinct groups based on the expression of tryptophan metabolism-related genes (Figure 5A). Furthermore, we examined the expression patterns of these genes within the different clusters. Notably, TDO2, HAAO, ACAT2, and DDC did not exhibit significant differences between the two groups (Figure 5B, 5C). Additionally, based on the results of principal component analysis (PCA), patients with varying risk profiles were successfully segregated into two distinct groups (Figure 5D). Moreover, we investigated the immune cell infiltration patterns in relation to the different clusters, shedding light on the immune microenvironment associated with each group (Figure 5E, 5F).

### Analysis of functional enrichments

Enrichment analysis was performed using the tryptophan metabolism-related genes. The molecular function (MF) analysis revealed their involvement in various functions, including transcription coregulator binding and histone arginine and methyltransferase activity. The biological processes (BP) associated with these genes encompassed diverse functions such as the regulation of cell migration, Aβ clearance, regulation of neurotransmitter transport, tryptophan catabolic process to kynurenine, serotonin biosynthetic process, and glycolytic process. Additionally, the cellular component (CC) analysis indicated their localization in the cytosol (Supplementary Figure 1A).

Pathway analysis further elucidated the functional implications of these genes. The enriched pathways included the chemokine signaling pathway, neuroactive ligand receptor interaction, p53 signaling pathway, apoptosis, tryptophan metabolism, and serotonergic synapse (Supplementary Figure 1B). These findings provide valuable insights into the molecular mechanisms and pathways associated with tryptophan metabolism in the context of Alzheimer’s disease.

### Building a co-expression network and module detection

To ensure a scale-free topology in the co-expression network, a soft-thresholding power was employed to establish an appropriate approximation (Supplementary Figure 2A). Subsequently, the genes with the highest variance were organized and integrated into seven distinct co-expression modules (Supplementary Figure 2B). To explore the relationship between module eigengenes and clinical characteristics, Pearson’s correlation analysis was performed (Supplementary Figure 2C). Notably, the turquoise module exhibited strong connections with the “Group” attribute, which represents the classification of AD and non-AD samples, indicating its significant association with the disease (Supplementary Figure 2D and Appendix 4). These findings shed light on the potential relevance of the turquoise module in Alzheimer’s disease.

### Clustering co-expression network construction and module detection

**Figure 6:**
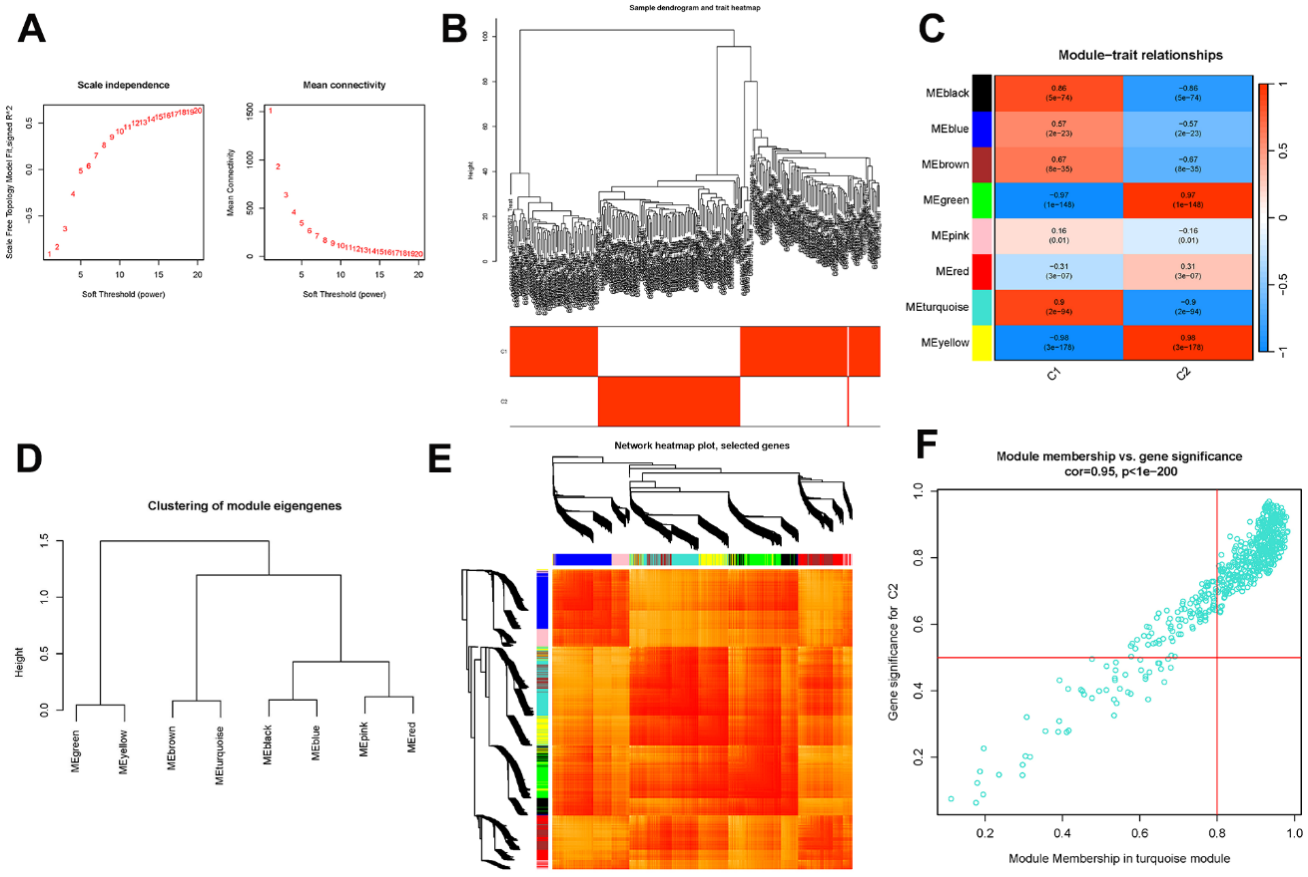
**Co-expression module construction.** (**A**) Soft threshold power mean connection and scale-free fitting index analysis. (**B**) Dendrogram clustering. (**C**) Heatmap of correlations between module eigengenes and clinical characteristics. (**D**) Gene scatterplot in the turquoise module. (**E**) Clustering of dendrograms according to dynamic tree cutting, the genes were sorted into distinct modules using hierarchical clustering with a threshold of 0.25. Each color represents a separate module. (**F**) Gene scatterplot in the turquoise module.

To establish a scale-free topology approximation for the network, a soft-thresholding power was applied, as depicted in Figure 6A. By clustering the genes based on their variance, co-expression modules were formed, as shown in Figure 6B. Pearson’s correlation analysis was then employed to investigate the relationship between module eigengenes and clinical characteristics, as illustrated in Figure 6C. Notably, the module exhibited strong connections with the “Group” attribute, representing the classification of AD and ND samples, indicating its significant association with the disease, as depicted in Figure 6D and (Appendix 5). The genes with the highest variance were further organized into eight co-expression modules, as presented in Figure 6E. Among these modules, the turquoise module demonstrated a high level of connectivity with the “Group” attribute, suggesting a strong association with AD and ND samples, as shown in Figure 6F.

### Developing a model for least absolute shrinkage and operator selection

**Figure 7:**
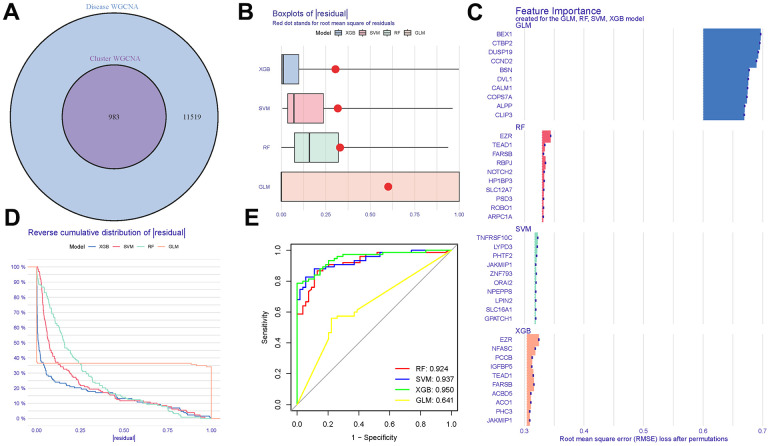
**Cluster construction of co-expression modules.** (**A**) Identification of tryptophan metabolism-related genes with a Venn diagram. (**B**, **C**) Residual expression patterns. (**D**) Model trend chart. (**E**) AUC of train group.

The intersection of DEGs, grey module genes (from WGCNA analysis), and tryptophan metabolism-related genes resulted in overlapping genes, as shown in Figure 7A and (Appendix 6). The boxplots illustrate the residual expression patterns of these genes in AD samples, highlighting the differences between AD and control groups, as presented in Figure 7B. Additionally, the proportions of the four different modes (DEGs, grey module genes, tryptophan metabolism-related genes, and overlapping genes) exhibit distinct variations, as depicted in Figure 7C. The expression of predictive values from the four models at different stages demonstrates noticeable differences, as shown in Figure 7D. Notably, the tryptophan metabolism-related genes exhibit a satisfactory diagnostic capacity in distinguishing AD from control samples, with area under the curve (AUC) values of RRF: 0.924, SVM: 0.937, XGB: 0.950, GLM: 0.641, as presented in Figure 7E. Among these models, the XGB model demonstrates the highest accuracy and stability (Appendix 7).

### Model validation

**Figure 8:**
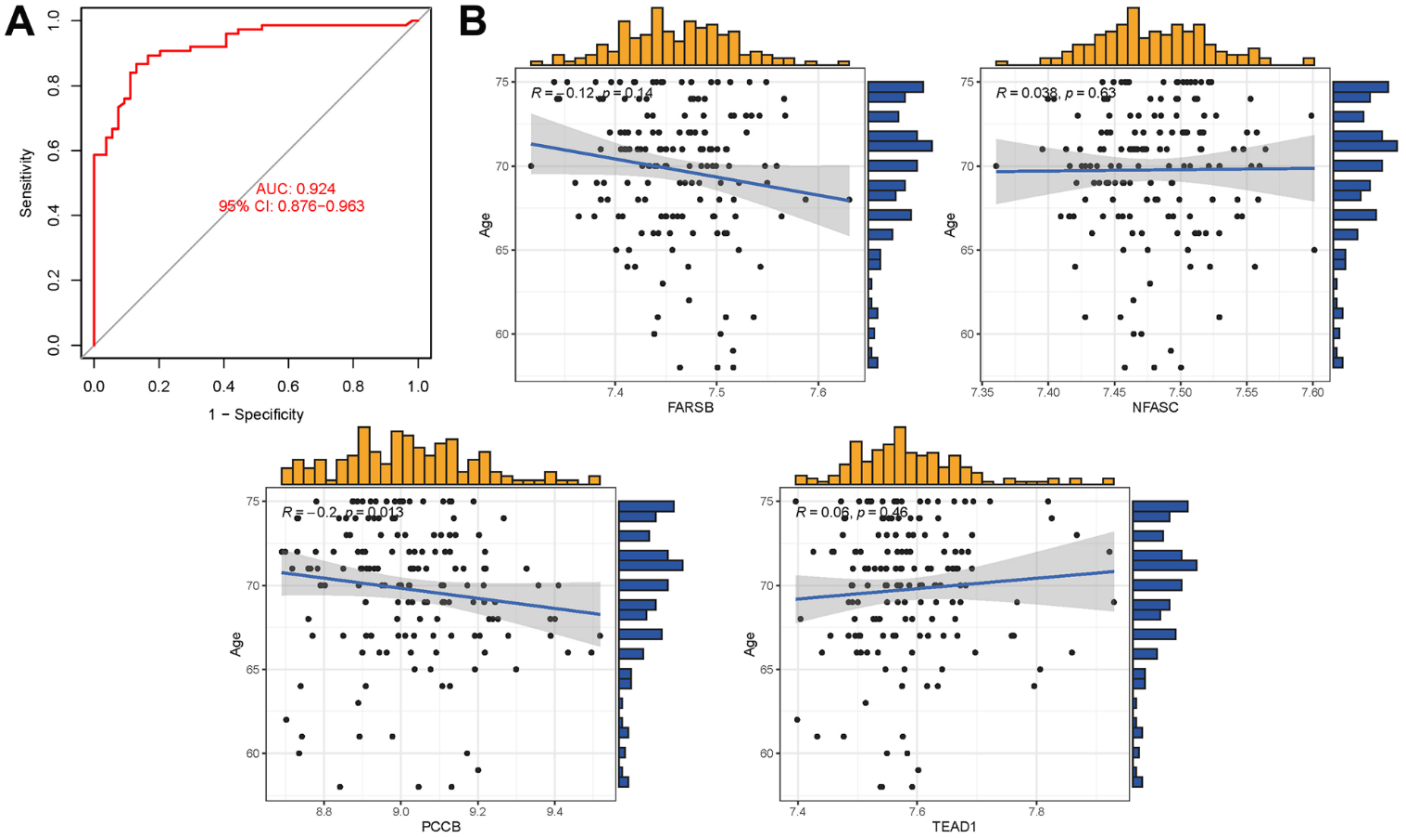
(**A**) AUC of test group. (**B**) Analysis of the relationship between hub genes and age.

In the external validation dataset GSE63060, the tryptophan metabolism-related genes exhibit an AUC of 0.924 (95% CI 0.876-0.963), indicating their potential as diagnostic markers for AD (Figure 8A). Additionally, the correlation analysis between the five hub genes and age revealed that PCCB and FARSB were negatively correlated with age, while NFASC and TEAD1 were positively correlated with age (Appendix 8). However, it is important to note that the P values for FARSB, NFASC, and TEAD1 were greater than 0.05, suggesting that these correlations may not be statistically significant (Figure 8B).

### Drug-gene interactions

According to the XGB model, one of the hub genes predicted three drugs, and one of these drugs is Dexamethasone (Table 2). Furthermore, we conducted predictions for all interacting genes associated with drug-gene relationships, and the results can be found in Appendix 9.

**Table 2:** Drug-gene interactions in the XGB model.

**Search term**	**Match term**	**Gene**	**Drug**	**Interaction types**	**Sources**
TEAD1	TEAD1	TEAD1	DEXAMETHASONE	Unknown	NCI

### Validation of the 5-gene signature in APP/PS1 transgenic mice

**Figure 9:**
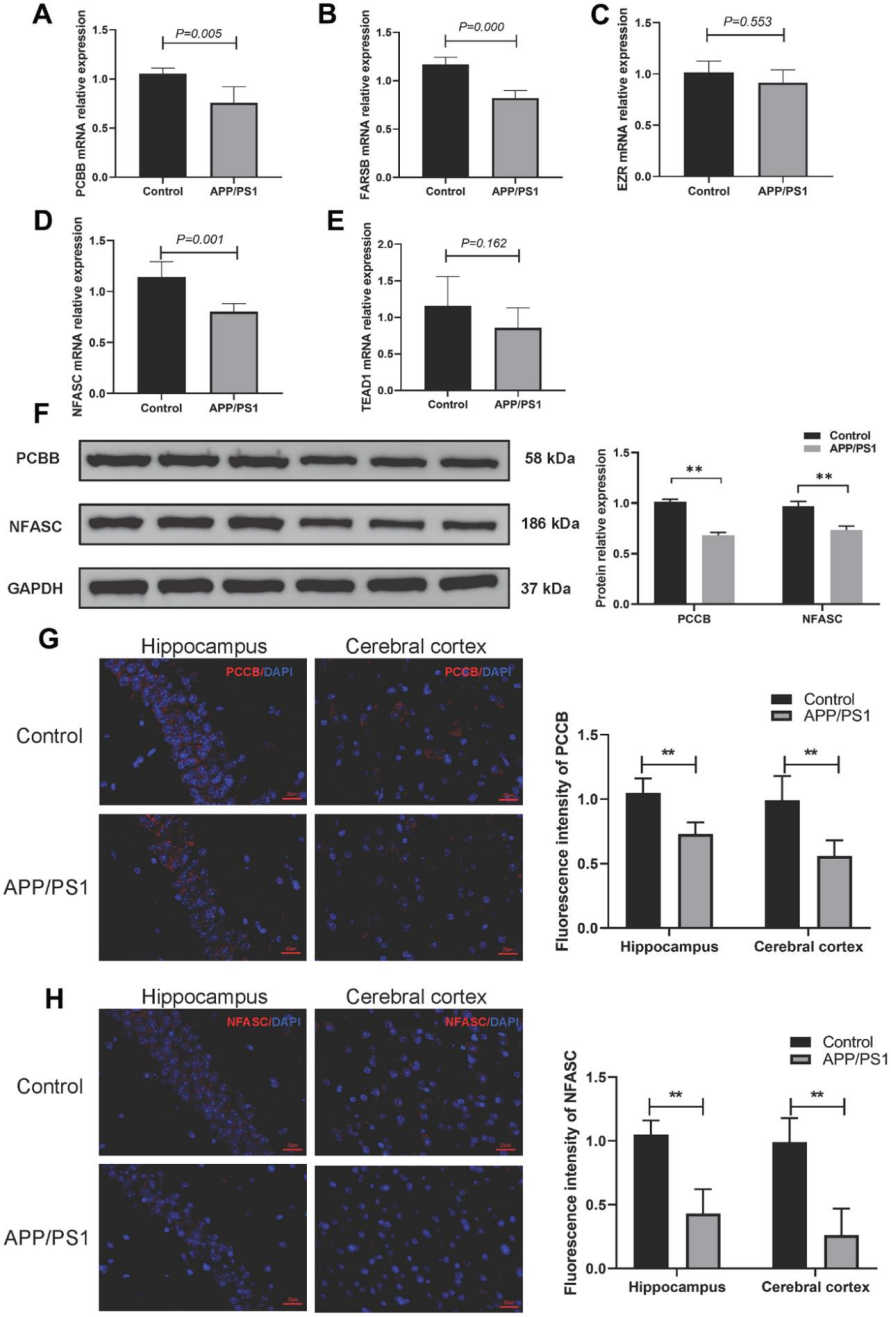
**Validation of 5 gene markers in APP/PS1 mice.** (**A**–**E**) qPCR to determine the mRNA expression of PCBB (**A**), FARSB (**B**), EZR (**C**), NFASC (**D**), and TEAD1 (**E**). (**F**) Western blotting to determine the protein expression levels of PCCB and NFASC. (**G**, **H**) Immunofluorescence to assess the expression of PCCB (**G**) and NFASC (**H**) in the hippocampus and cerebral cortex.

The qPCR analysis revealed that the mRNA levels of PCCB and NFASC were down-regulated in the AD group compared to the control group, which is consistent with the previous analysis. However, the expression profile of FARSB did not align with the previous results. Furthermore, there were no significant differences in the expression levels of TEAD1 and EZR between the AD group and the wild-type group (Figure 9A–9E). The Western blotting results (Figure 9F and Supplementary Figure 3) demonstrated that the expression of PCCB and NFASC in the cerebral cortex of APP/PS1 transgenic mice was significantly decreased compared to the wild-type group. Additionally, immunofluorescence staining showed decreased expression of PCCB and NFASC in the cerebral cortex and hippocampus of APP/PS1 transgenic mice (Figure 9G–9H).

## DISCUSSION

Recent scientific investigations have amassed a substantial body of research evidence highlighting the prevalent metabolic disorders observed in AD, with multiple disruptions in metabolic pathways considered as potential pathogenic factors [16]. Unraveling the intricate relationship between AD and metabolic disorders assumes paramount importance in identifying novel targets for disease treatment. Notably, neurons in AD-afflicted brains are known to exhibit profound deficits in glucose metabolism, thereby suggesting that alternative energy sources could potentially mitigate disease-specific neuronal death [17]. Moreover, studies have indicated significant alterations in the levels of several amino acids in the brains and plasma of AD patients, indicating that perturbations in amino acid metabolism may play a pivotal role in driving AD progression [18].

Of particular interest as an intriguing regulatory node, tryptophan metabolism undergoes dynamic changes under various pathological conditions, attracting considerable attention from researchers. A comprehensive metabolomic study focusing on plasma and cerebrospinal fluid (CSF) analysis of AD subjects revealed reduced levels of tryptophan in both CSF and plasma among individuals with mild cognitive impairment [19]. Tryptophan and its associated metabolites can inhibit various enzymes participating in the biosynthesis of Aβ, and one metabolite, 3-hydroxyanthranilate, can directly inhibit neurotoxic Aβ oligomerization; however, whilst certain trp metabolites are neuroprotectant, other metabolites, such as quinolinic acid, are directly toxic to neurons and may themselves contribute to AD progression. Tryptophan metabolites also can influence microglia and associated cytokines to modulate the neuroinflammatory and neuroimmune factors that trigger pro-inflammatory cytotoxicity in AD [6]. Notably, melatonin, a product of tryptophan metabolism in the serotonin pathway, has demonstrated increased clearance rates of amyloid beta and noteworthy immunomodulatory effects [20]. Furthermore, KYNA, a product of tryptophan metabolism through the kynurenine pathway and synthesized and released by astrocytes in the brain, acts as an antagonist for N-methyl-D-aspartic acid (NMDA) and α7 nicotinic acetylcholine (α7nACh) receptors, exhibiting potential neuroprotective effects [21]. The kynurenine pathway is responsible for more than 95% of tryptophan catabolism. Its initial and rate-limiting step involves the conversion of tryptophan to kynurenine by the enzymes indoleamine 2,3-dioxygenase (IDO) and tryptophan 2,3-dioxygenase (TDO) [22], which produce neuroactive and anti-inflammatory metabolites [23] and also play a role in anti-inflammatory immune signaling pathways. Age-related Kyn pathway activation might contribute to AD pathology in humans, and inhibition of TDO was found to reduce AD-related cellular toxicity and behavioral deficits in animal models. Moreover, TDO inhibition reversed recognition memory deficits without producing measurable changes in cerebral Kyn metabolites. TDO inhibition did not affect spatial learning and memory or anxiety-related behavior. These data indicate that age-related Kyn pathway activation is not specific for humans and could represent a cross-species phenotype of aging [23]. Consequently, investigating the implications of these pathways in neurodegenerative disorders such as AD holds significant scientific value [22]. The kynurenine pathway and thioredoxin-interacting protein (TXNIP) activity regulate inflammation and neurotoxicity in AD. There was a causal relationship among epigenomic state, TXNIP expression, cerebral-spleen tryptophan metabolism, inflammatory cytokine production, and cognitive decline; and they provide a potential mechanism for Txnip gene regulation in normal and pathologic conditions, suggesting TXNIP levels may be a useful predictive or diagnostic biomarker for Aβ_40_/Aβ_42_ targeted AD therapies [24]. Moreover, immune cells within the central nervous system actively participate in the tryptophan metabolic pathway. Astrocytes catalyze the conversion of kynurenine (KYN) to kynurenic acid (KYNA) through the action of kynurenine aminotransferases (KATs), while microglia convert KYN to 3-hydroxykynurenine (3-HK) via kynurenine monooxygenase (KMO) [6]. These metabolites, in turn, influence the activation of microglia and astrocytes involved in the regulation of neuroinflammation and neuroimmune factors in AD [25]. These pieces of evidence collectively underscore the intimate association between tryptophan metabolism and AD progression.

Meanwhile, several recent studies have underscored the significance of TrpMgs in neurodegenerative diseases. Fifita et al. conducted whole-genome sequencing of 614 cases of sporadic amyotrophic lateral sclerosis (ALS) and identified 4 TrpMgs that may serve as risk factors for ALS through alterations in the kynurenine pathway and subsequent neuroinflammation [26]. Furthermore, George Anderson et al. reviewed the interplay between tryptophan metabolism and immune-inflammation gene interactions in the context of Parkinson’s neurodegeneration [27]. However, the precise pathophysiological role of tryptophan metabolism-related genes in AD development remains unclear. In this study, we obtained data from AD patients in the GEO database and integrated it with TrpMgs. Through differential analysis and a risk model, we identified TrpMgs associated with the prognosis of AD patients. These findings hold promise in facilitating drug development for AD and provide novel avenues for therapeutic interventions.

Our analysis detected 17 DEGs associated with tryptophan metabolism in AD. To delve deeper into the role of TrpMgs in AD, we identified Trp-metabolism DEGs by intersecting DEGs, conducting WGCNA, and focusing on TrpMgs. These genes primarily concentrate on pathways associated with the disturbance of immune-inflammatory balance in the nervous system. GO and KEGG enrichment analyses suggest a pivotal role of neuroinflammation in AD, including pathways such as the chemokine signaling pathway, p53 signaling pathway, and apoptosis pathway. Furthermore, using lasso logistic regression and XGBoost, two machine learning methods for variable selection, we identified five hub TrpMgs (PCCB, TEAD1, FARSB, NFASC, and EZR). Their diagnostic capacity was validated using external datasets, indicating their potential involvement in the AD process. For instance, PCCB is a mitochondrial enzyme involved in the catabolism of branched amino acids, such as isoleucine, threonine, methionine, and valine. Aberrant methylation of PCCB has been associated with epilepsy development by causing mitochondrial dysfunction [28]. Recent studies have also implicated PCCB as a potential marker for immune balance regulation in AD [29]. Another hub gene, TEAD1, is a transcription factor that plays a critical role in the Hippo signaling pathway, regulating homeostasis by influencing cell proliferation and apoptosis [30]. TEAD1 affects the generation and migration of cortical neurons through the regulation of the Hippo signaling pathway. Notably, TEAD1 is known to interact with ApoE, a key player in AD pathology and ApoE antibody inhibits Aβ-associated tau seeding and spreading in a mouse model [31], suggesting that TEAD1 may contribute to AD pathology through ApoE [32]. FARSB is implicated in human phenylalanine synthesis, and mutations in the FARSB gene have been associated with neurodevelopmental disorders affecting the brain, liver, and lungs [33]. However, concrete evidence linking FARSB to AD development is still lacking. NFASC is a transmembrane protein that may be involved in various processes such as neurite elongation, axon guidance, synaptogenesis, myelination, and neuron-glial cell interactions. NFASC levels were significantly decreased in the cerebrospinal fluid of AD patients [34], which may be related to the loss of dendritic spines in hippocampal neurons [35]. EZR is a cytoplasmic peripheral membrane protein involved in the regulation of axon growth through interactions with the actin cytoskeleton [36]. Bara et al. reported that BACE1, a major drug target for AD, plays a crucial role in semaphorin 3A axonal guidance of hippocampal and thalamic neurons. The process involves BACE1 generating active membrane-binding proteins to cleave CHL1 fragments and relay Sema3A signals to the cytoskeleton via the ezrin-radixin-moesin pathway [37].

Furthermore, we investigated and analyzed the correlation between hub genes and age. Pearson correlation coefficient analysis revealed that PCCB, FARSB, NFASC, and TEAD1 exhibited correlations with age. Notably, PCCB demonstrated a statistically significant association with age, with a p-value less than 0.05. Although there is no direct evidence of PCCB’s involvement in tryptophan metabolism, it is a mitochondrial enzyme involved in the catabolism of numerous amino acids [28]. Exploring the impact of PCCB on the tryptophan metabolism pathway in AD poses an intriguing question that warrants further investigation. Moreover, we validated the mRNA levels of these genes in the hippocampus of APP/PS1 transgenic mice using qPCR. The results indicated that FARSB, PCCB, and NFASC were downregulated compared to the control group, while TEAD1 and EZR did not show significant differences between the AD and control groups. The contrasting outcomes observed *in vitro* and *in vivo* may be attributed to species differences. Additionally, we assessed the protein expression of PCCB and NFASC in the hippocampus of AD mice and found significantly lower levels compared to the control group, consistent with previous reports [29]. In our previous study [14], the result of MWM showed learning and memory deficits in APP/PS1 mice. Combining the expressions mRNAs and proteins of PCCB and NFASC in the hippocampus of APP/PS1 mice, the result suggested there are positive relation between those genes and the cognitive functions. The down-regulation of PCCB and NFASC results in learning and memory deficits. Collectively, these findings shed light on key pathogenic features of AD that warrant further investigation.

Current studies have demonstrated a close association between peripheral immune dysfunction, brain immune environment imbalance, and the occurrence of AD. Neuroinflammation is now considered the initiating factor of AD and the pathological basis for disease progression [38]. Imbalances in the synthesis and release of proinflammatory and anti-inflammatory cytokines in response to disease-related molecular patterns contribute to the sustained development of neuroinflammation [39]. To successfully alleviate chronic neuroinflammation in AD and restore neuronal function, the identification of unique diagnostic biomarkers and therapeutic targets from the perspective of neuro-immune interaction is crucial for both basic and clinical research on AD. Our results indicated that memory B cells, T follicular helper cells, M0 macrophages, and activated dendritic cells exhibited reduced infiltration in AD, while prototic B cells, CD8T cells, and neutrophils exhibited increased infiltration. AD transgenic mouse models lacking specific adaptive immune populations show higher amyloid-beta deposition and more severe neuroinflammation [40]. Therefore, our findings contribute to the understanding of pathological impairment and cognitive decline caused by immune dysregulation in AD. Additionally, we discussed the expression of TrpMgs in the immune microenvironment. The findings revealed high expression of activated B cells, plasma cells, CD8T cells, activated CD4T cells, macrophage M0, and neutrophils in cluster 1. Cluster 2 exhibited high expression of CD4T cells memory resting, regulatory T cells (Tregs), NK cells activated, monocytes, macrophage M2, and eosinophils. These findings further support the notion that the pathogenesis of TrpMgs in AD is closely related to inflammation and immune response.

Despite the valuable insights gained from this study, several limitations should be acknowledged. Firstly, the data utilized in this study were obtained from the GEO database, and therefore, the quality and reliability of the data require further validation, though having compared the data before analysis. Secondly, the sample size needs to be expanded to enhance statistical power. Thirdly, the utilization of mice instead of human tissue for gene screening warrants further validation. Finally, the specific pathways through which hub genes regulate tryptophan metabolism in AD need to be elucidated through additional research endeavors, and the hypothesis needs to be further proved with more direct evidence. Additionally, the study focuses on mRNAs associated with Try metabolism, so there is limited understanding of the underlying mechanisms involved.

## CONCLUSIONS

In conclusion, immune disorders leading to pathological damage and cognitive impairment are implicated as potential etiological factors in AD. The regulation of AD immune-inflammatory response involves TrpMgs. Therefore, a promising therapeutic approach for AD could involve targeting tryptophan metabolism in combination with immunotherapy. Through comprehensive screening, PCCB, TEAD1, FARSB, NFASC, and EZR have been identified as potential diagnostic markers and therapeutic targets for AD. Experimental validation in the cerebral cortex and hippocampus of APP/PS1 transgenic mice confirmed the downregulated expression of PCCB and NFASC. Notably, tryptophan metabolism-related genes, namely PCCB and NFASC, exhibit considerable diagnostic value in distinguishing AD from other conditions. These findings emphasize the potential significance of exploring tryptophan metabolism and immune-inflammatory responses in the development of therapeutic interventions and diagnostic strategies for AD. Further investigations are warranted to elucidate the underlying mechanisms and translate these findings into clinical applications.

## Supplementary Material

Supplementary Figures

Appendix 1

Appendix 2

Appendix 3

Appendix 4

Appendix 5

Appendix 6

Appendix 7

Appendix 8

Appendix 9
