# Supplementary material for: Identification of tryptophan metabolism-related genes in immunity and immunotherapy in Alzheimer’s disease
Source: Aging (Albany NY). 2023 Nov 20;15(22):13077–99. doi: 10.18632/aging.205220 (PMC10713402; doi:10.18632/aging.205220)
Supplement: Supplementary Figures [file aging-15-205220-s001.pdf]

SUPPLEMENTARY FIGURES

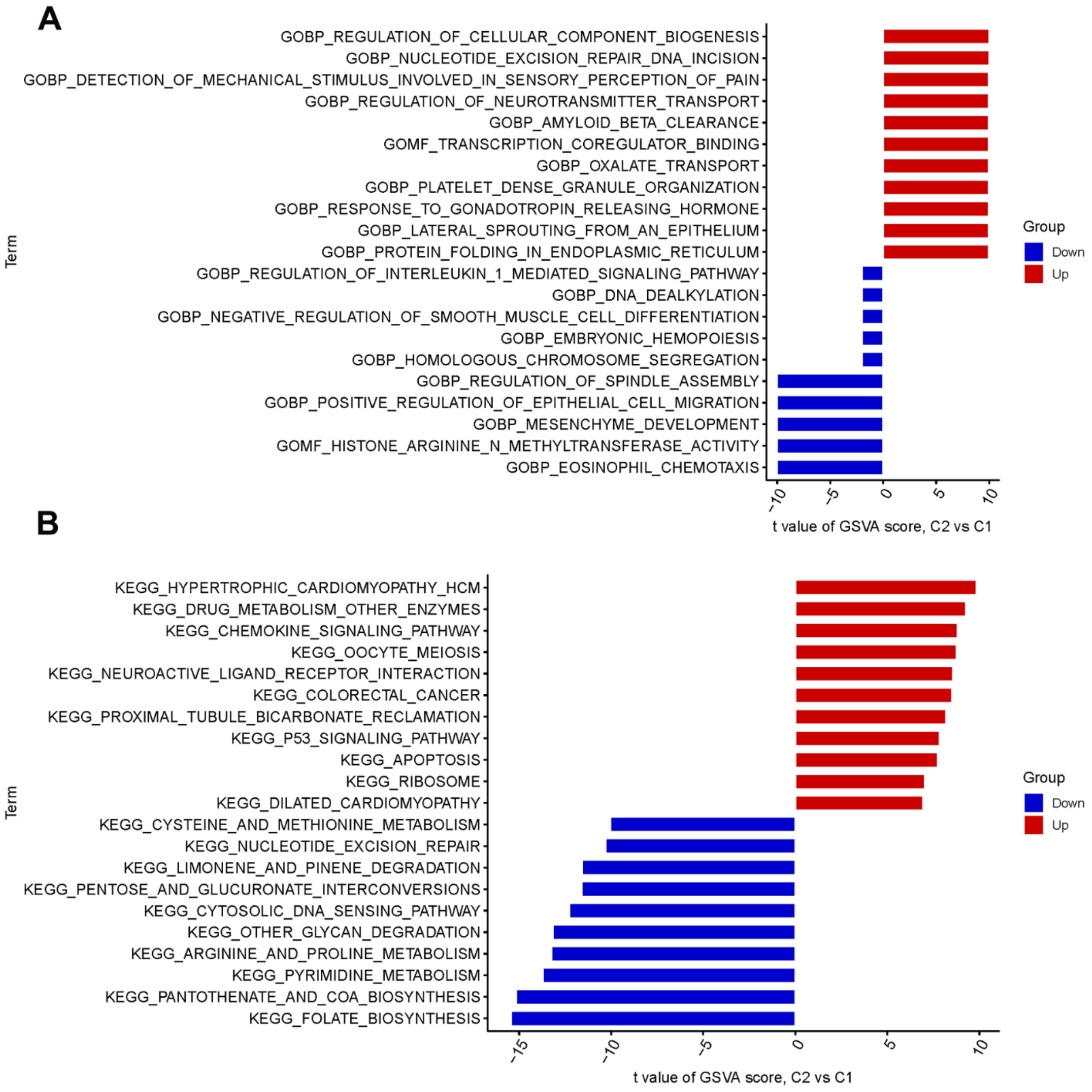

Supplementary Figure 1. Enrichment analysis for DEGs. (**A**) GO. (**B**) KEGG. (**A**) Barplot graph for GO enrichment (the longer bar means the more genes enriched; q-value: the adjusted p-value). (**B**) Barplot graph for KEGG pathways (the longer bar means the more genes enriched).

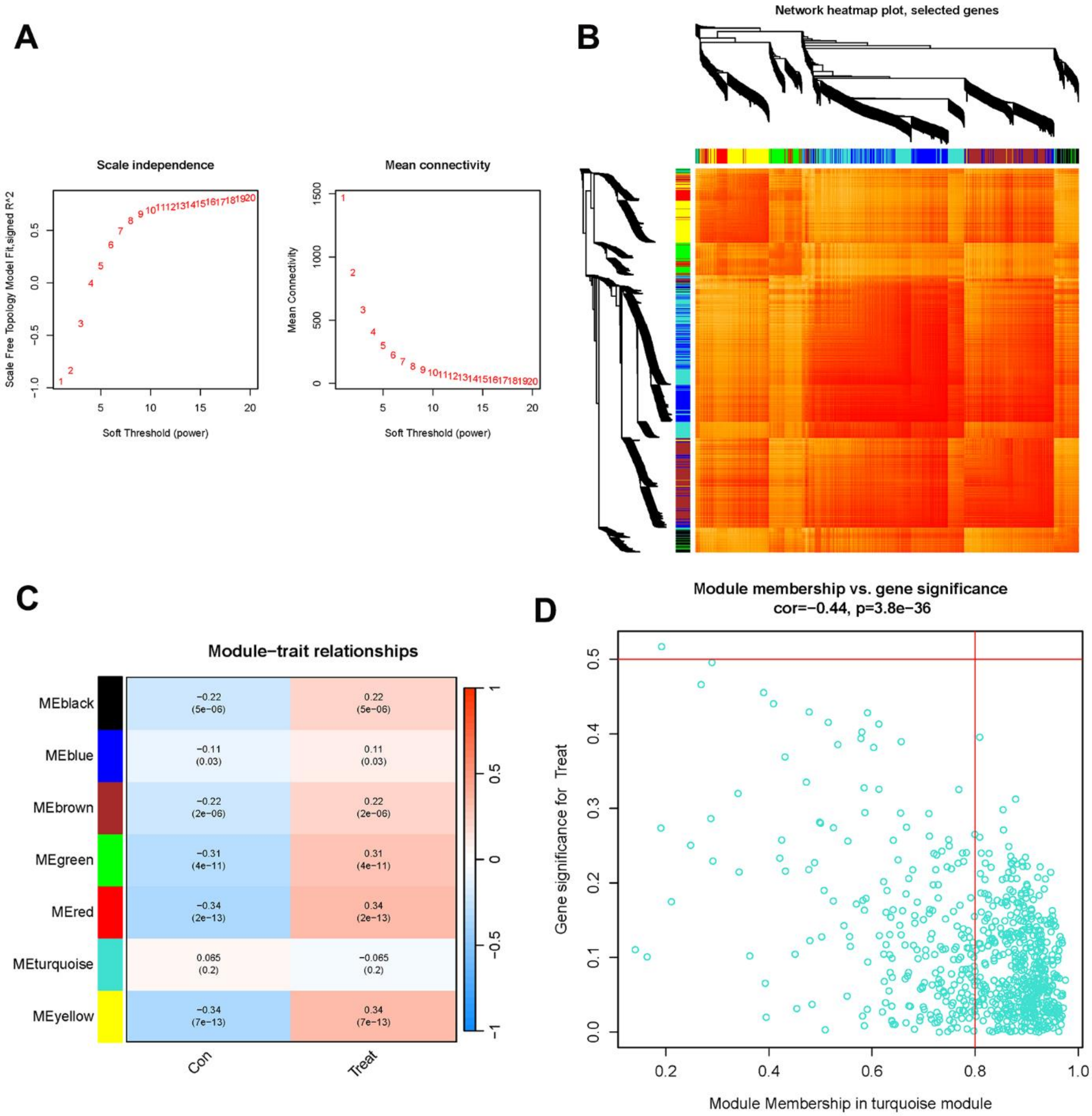

Supplementary Figure 2. Co-expression module construction. (**A**) Soft threshold power mean connection and scale-free fitting index analysis. (**B**) Clustering of dendrograms according to dynamic tree cutting, the genes were sorted into distinct modules using hierarchical clustering with a threshold of 0.25. Each color represents a separate module. (**C**) Heatmap of correlations between module eigengenes and clinical characteristics. (**D**) Gene scatterplot in the turquoise module.

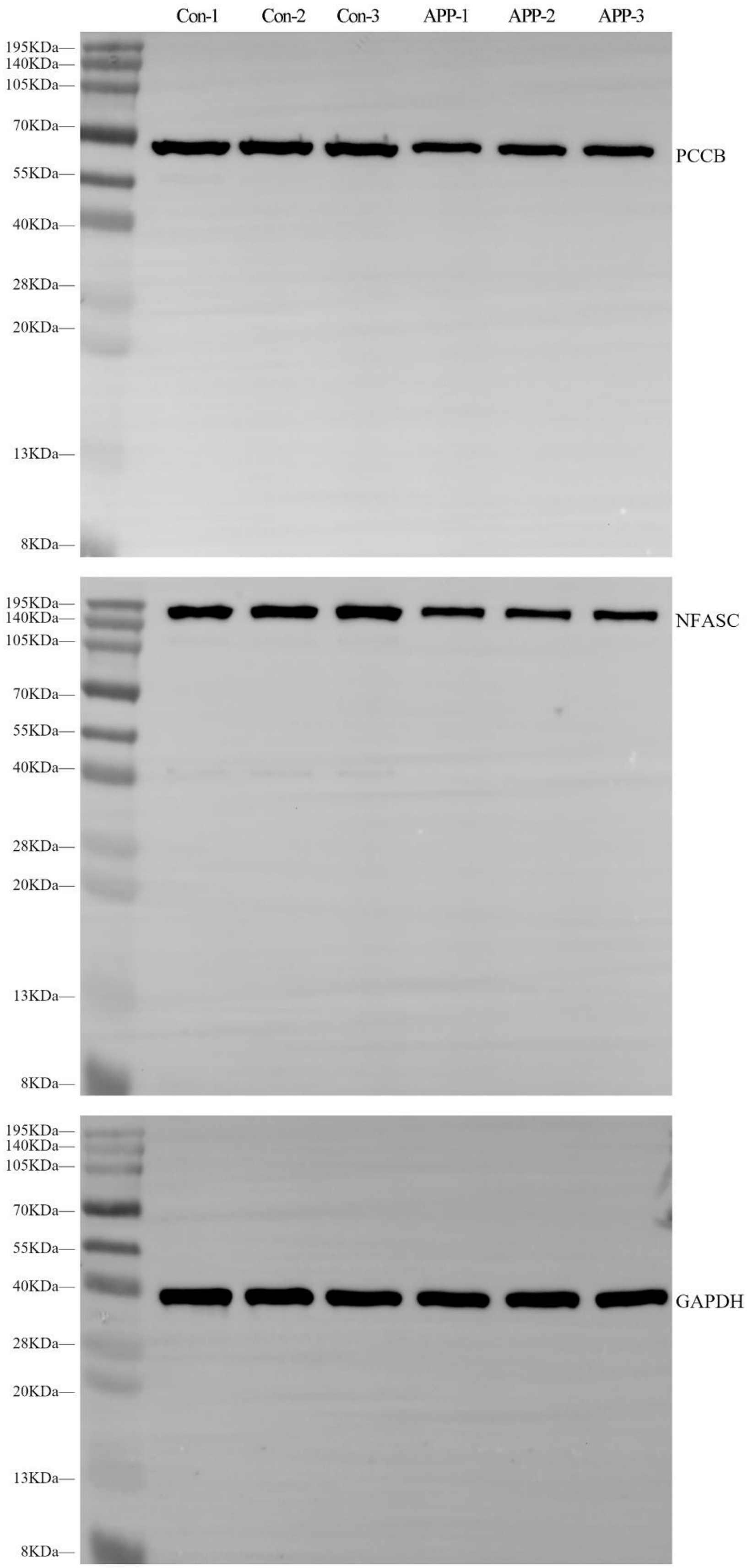

Supplementary Figure 3. The original image of Western blotting.
