# Supplementary material for: Identification of tryptophan metabolism-related genes in immunity and immunotherapy in Alzheimer’s disease
Source: Aging (Albany NY). 2023 Nov 20;15(22):13077–99. doi: 10.18632/aging.205220 (PMC10713402; doi:10.18632/aging.205220)
Supplement: Appendix 1 [file aging-15-205220-s002.docx]

# Appendix 1. Datasets and tryptophan metabolism.

**Table 1. Tryptophan metabolism genes**

| IDO1 | GCDH | TPH2 | AOX1 |
| --- | --- | --- | --- |
| TDO2 | HADHA | TPH1 | ASMT |
| IDO2 | EHHADH | DDC | AANAT |
| AFMID | ECHS1 | MAOB | CYP1A1 |
| KMO | HADH | MAOA | CYP1A2 |
| KYNU | ACAT2 | ALDH2 | CYP1B1 |
| HAAO | ACAT1 | ALDH3A2 | INMT |
| ACMSD | KYAT3 | ALDH1B1 | IL4I1 |
| OGDHL | KYAT1 | ALDH7A1 | AOC1 |
| OGDH | AADAT | ALDH9A1 | CAT |
