# Supplementary material for: Identification of tryptophan metabolism-related genes in immunity and immunotherapy in Alzheimer’s disease
Source: Aging (Albany NY). 2023 Nov 20;15(22):13077–99. doi: 10.18632/aging.205220 (PMC10713402; doi:10.18632/aging.205220)
Supplement: Appendix 2 [file aging-15-205220-s003.docx]

# Appendix 2. DEGs linked to tryptophan metabolism genes.

**Table 2. 17 DEGs linked to tryptophan metabolism genes.**

| ID | GSM119615 | GSM119616 | GSM119617 | GSM119618 | GSM119619 |
| --- | --- | --- | --- | --- | --- |
| TDO2 | 3.993636 | 3.917518 | 4.122374 | 2.816972 | 4.65514 |
| IDO2 | 2.547292 | 4.409812 | 4.084605 | 3.384879 | 3.349799 |
| KYNU | 3.41152 | 3.497331 | 3.613856 | 3.758062 | 2.808747 |
| HAAO | 4.682708 | 4.440715 | 4.963799 | 4.772167 | 4.110601 |
| OGDHL | 5.954859 | 6.392547 | 6.236326 | 6.111209 | 6.093868 |
| OGDH | 3.91503 | 4.457656 | 4.308076 | 4.428794 | 4.409304 |
| EHHADH | 4.491764 | 4.284879 | 4.457656 | 4.5466 | 4.747997 |
| ACAT2 | 5.780492 | 5.503615 | 4.900442 | 5.373276 | 6.033585 |
| ACAT1 | 5.642894 | 5.022368 | 5.02485 | 4.678478 | 5.641497 |
| AADAT | 4.153802 | 4.924491 | 4.942866 | 5.045962 | 4.785313 |
| TPH2 | 2.780285 | 4.000447 | 5.027119 | 4.501129 | 3.834479 |
| DDC | 3.894608 | 3.946489 | 3.047707 | 3.174551 | 3.796567 |
| MAOB | 5.793772 | 5.823652 | 4.974145 | 5.485081 | 5.817374 |
| AOX1 | 4.537604 | 5.034124 | 5.144452 | 4.700085 | 5.141151 |
| CYP1A1 | 3.184365 | 3.360679 | 5.402973 | 4.710225 | 3.704775 |
| CYP1A2 | 4.424855 | 4.640611 | 5.296096 | 4.802325 | 4.478731 |
| INMT | 2.781055 | 4.13843 | 3.572934 | 3.971955 | 4.562483 |
