# Supplementary material for: Identification of tryptophan metabolism-related genes in immunity and immunotherapy in Alzheimer’s disease
Source: Aging (Albany NY). 2023 Nov 20;15(22):13077–99. doi: 10.18632/aging.205220 (PMC10713402; doi:10.18632/aging.205220)
Supplement: Appendix 3 [file aging-15-205220-s004.docx]

# Appendix 3. Chromosomal positions of tryptophan metabolism-related genes.

**Table 3. chromosomal positions of tryptophan metabolism-related genes.**

| Chromosome | chromStart | chromEnd | Gene |
| --- | --- | --- | --- |
| chr1 | 165662216 | 165698863 | ALDH9A1 |
| chr1 | 241532134 | 241595642 | KMO |
| chr2 | 26190635 | 26244726 | HADHA |
| chr2 | 38066973 | 38109902 | CYP1B1 |
| chr2 | 42767089 | 42792593 | HAAO |
| chr2 | 134838547 | 134902034 | ACMSD |
| chr2 | 142877498 | 143055832 | KYNU |
| chr2 | 200585868 | 200677064 | AOX1 |
| chr3 | 185190624 | 185281990 | EHHADH |
| chr4 | 107989714 | 108035175 | HADH |
| chr4 | 155854738 | 155920406 | TDO2 |
| chr4 | 170060222 | 170091699 | AADAT |
| chr5 | 126541841 | 126595418 | ALDH7A1 |
| chr6 | 159760328 | 159779055 | ACAT2 |
| chr7 | 30697985 | 30757602 | INMT |
| chr7 | 44606572 | 44709066 | OGDH |
| chr7 | 50458436 | 50565457 | DDC |
| chr7 | 150824627 | 150861504 | AOC1 |
| chr8 | 39902275 | 39928444 | IDO1 |
| chr8 | 39934614 | 40016391 | IDO2 |
| chr9 | 38392664 | 38398661 | ALDH1B1 |
| chr10 | 49734643 | 49762379 | OGDHL |
| chr10 | 133362480 | 133373689 | ECHS1 |
| chr11 | 18017564 | 18042426 | TPH1 |
| chr11 | 34438925 | 34472062 | CAT |
| chr11 | 108121516 | 108147776 | ACAT1 |
| chr12 | 71938846 | 72186618 | TPH2 |
| chr12 | 111766887 | 111817529 | ALDH2 |
| chr15 | 74719542 | 74725610 | CYP1A1 |
| chr15 | 74748844 | 74756202 | CYP1A2 |
| chr17 | 19648136 | 19677598 | ALDH3A2 |
| chr17 | 76453351 | 76470117 | AANAT |
| chr17 | 78187317 | 78207701 | AFMID |
| chr19 | 12891026 | 12914207 | GCDH |
| chr19 | 49889654 | 49929539 | IL4I1 |
| chrX | 1615001 | 1643081 | ASMT |
| chrX | 43654907 | 43746824 | MAOA |
| chrX | 43766611 | 43882447 | MAOB |
