# Supplementary material for: Identification of tryptophan metabolism-related genes in immunity and immunotherapy in Alzheimer’s disease
Source: Aging (Albany NY). 2023 Nov 20;15(22):13077–99. doi: 10.18632/aging.205220 (PMC10713402; doi:10.18632/aging.205220)
Supplement: Appendix 4 [file aging-15-205220-s005.docx]

# Appendix 4. Co-expression network construction and module detection.

**Table 4. Co-expression Network Construction and Module Detection.**

| SCP2 | RBAK | FOXG1 | DMXL1 | PSMB10 | NPTN |
| --- | --- | --- | --- | --- | --- |
| SMAD2 | CA8 | SLCO2B1 | LTBP1 | MAL2 | CEP97 |
| NHLH2 | AEBP1 | RASA2 | IMPA2 | PCDHB7 | NEK5 |
| RIMS4 | RNF31 | LIF | ARMCX6 | C20orf27 | IARS |
| RHOT1 | CSNK1G2 | GATAD2B | DUSP23 | MSI1 | COBLL1 |
| HUWE1 | HPS6 | BTBD3 | TPM2 | ROBO1 | TMEM145 |
| DUOX2 | SLC20A2 | YAF2 | KCNK12 | VIM | BST2 |
| KRT77 | NDE1 | VAPA | SNRNP200 | METAP2 | PODNL1 |
| GINS1 | TAS2R38 | RASGRP1 | ILF2 | PAIP1 | TMTC3 |
| KCNH4 | PTPN1 | SDHAF2 | FAIM2 | ANP32A | GAS2L1 |
| DNMBP | KCNA6 | FAM47E | NEFM | NCOA2 | SMCR8 |
| ERC2 | SLC10A4 | SETD7 | NPY1R | DKK3 | RANGAP1 |
| IFIH1 | PPP1R3C | RTN4 | HES6 | SCN10A | LHPP |
| XK | CRB3 | MAP6D1 | HK1 | TRIM37 | SLC6A13 |
| MFHAS1 | SEC11C | MTMR12 | EGR3 | NEFH | BAIAP2L2 |
| BRI3BP | TTTY12 | TMEM167A | OLFM3 | BTBD16 | PRKAR2A |
| CNGA1 | CCDC136 | HCST | OSTF1 | GRAMD1B | TP53I13 |
| SS18 | NEFL | CHORDC1 | TAF11 | FGFRL1 | SERPINH1 |
| INPP4A | KLK6 | C21orf91 | KLF10 | FNDC3B | SST |
| APBA2 | F8 | NEUROD6 | CFH | NENF | CAMK2D |
| STYK1 | ATG16L1 | MYH3 | VAMP5 | ZWINT | POLR2A |
| LSM3 | BTBD8 | ARF6 | AK5 | TUBA1A | CYFIP1 |
| CLPTM1 | SFXN5 | AMIGO2 | S100A12 | CROCC | NRXN3 |
| RFX3 | ARHGEF10 | LENEP | ZNF703 | CDC42SE1 | TOP1 |
| LMO3 | KY | ASB14 | IFT80 | SMURF2 | ATCAY |
| SIVA1 | STT3B | C16orf46 | GAK | TTYH3 | RPL11 |
| PCDHB12 | GPR157 | IGSF9B | CBS | CNTN4 | MAPK8IP1 |
| KCNJ13 | KRT82 | HPDL | EPHX1 | TNFRSF13B | NAP1L2 |
| SON | HIST1H2AC | UXS1 | LRRTM2 | PFN2 | ZNF554 |
| PHF1 | ZNF449 | MGST3 | EMP1 | MICAL2 | SLC22A3 |
| ALDH1L1 | BAMBI | DNAJB6 | FAM35A | PCSK2 | NACAP1 |
| SYT14 | OR6B1 | SEH1L | AP3S1 | RANBP9 | ARHGDIB |
| PASD1 | DACT3 | KPTN | CLSTN1 | SGCA | TMEM59L |
| LTA | NDUFB7 | COPE | HS6ST2 | MAST1 | LNPEP |
| OSCP1 | WDR3 | GABRR2 | LCN15 | TAGLN3 | OPHN1 |
| CNIH2 | C1orf105 | ZNF519 | ALAS1 | ENTPD4 | DYNLT3 |
| TSTD2 | HOXC12 | GTPBP10 | C14orf80 | NLN | IFITM3 |
| HIST1H4G | LCORL | SLC12A9 | ZNF711 | SPI1 | GPR26 |
| CD200 | LFNG | SACS | SRFBP1 | PITPNB | DPY19L2P2 |
| DMRTC2 | HRC | UCHL1 | RAB39B | CRTAC1 | ZNF296 |
| ECHDC2 | CYP8B1 | CFD | CD163 | GRIN2C | KCNA1 |
| NXPH3 | S100A6 | KIAA0232 | VGF | NCAM2 | RAB6A |
| PPL | NDUFS4 | MMEL1 | CLTB | KDM2B | DRD4 |
| BRMS1L | ZNF425 | CDC42BPB | SPCS3 | ACVR1B | GPR27 |
| EIF2S2 | ZNF645 | SV2A | GDAP1 | GABRA4 | TRAPPC1 |
| AP4S1 | MCM3 | GNG8 | APOBEC4 | SOX1 | AHRR |
| NR4A1 | NF1 | PAPPA2 | LRRC52 | TCTN2 | RFK |
| CYP2A7 | RASA1 | ZNF668 | WBSCR17 | RENBP | ZIC1 |
| PRSS33 | OSBPL6 | NOTCH1 | C4orf33 | MRAP2 | EVI5L |
| RGS8 | CCDC84 | ADIG | ACY1 | RICTOR | CD99L2 |
| PGD | ACTG2 | NCSTN | ZNF528 | PRKAA2 | CCNL2 |
| SEMA6D | EPC1 | AMPD3 | IL32 | VOPP1 | ZNF48 |
| OR7C1 | NFE2 | HIBADH | C15orf52 | GPR85 | SYNGR3 |
| DNTTIP1 | EMP3 | SLK | DUSP22 | FGF14 | HIPK3 |
| AAK1 | PPARG | B9D2 | NRN1 | PLEKHA4 | SLC2A13 |
| GIN1 | GALNT1 | TMEM119 | DAB2 | CNKSR2 | MEIS2 |
| MYOM3 | DDX19B | SNX9 | FAM181B | STARD4 | DDX51 |
| FAM65C | SCRG1 | HCFC1R1 | FLRT3 | FSCN1 | KIRREL3 |
| CHMP1B | GPATCH1 | KIAA1683 | GATC | FEM1B | TXLNA |
| STK4 | GATS | MCC | PLCB3 | RALGAPA2 | DPYSL3 |
| HIST1H3F | S100A10 | VPS13C | FOLH1 | BLOC1S2 | ZBTB41 |
| PIK3R2 | SOX8 | DGKH | IL13 | SLC9A6 | ZDHHC8 |
| PRKCH | ADORA2B | TSPAN1 | KIF9 | ARL6 | HSPB1 |
| CNTFR | HTR2C | RTKN | FAM134A | LY6E | SYT1 |
| PQLC3 | OGN | PIK3CB | ZBTB26 | HGS | CADM4 |
| CD74 | KCNA4 | CHRM3 | CD44 | ANK3 | BEX1 |
| TGFBR1 | PNMAL1 | ITFG2 | IKBKAP | DNAJC1 | GPRASP1 |
| AAGAB | CNRIP1 | RXFP1 | SERINC5 | ISG20 | ZNF121 |
| SH3PXD2A | FIGN | HEY1 | LCAT | MFAP4 | NOS3 |
| SYT6 | IFNA10 | MESDC2 | APOBEC2 | ITM2B | MGP |
| ZNF488 | LPIN2 | ZSCAN29 | LYPD1 | XPOT | TAF6L |
| P2RY12 | FAM71D | SLC35D1 | PDPK1 | LIME1 | SCN3A |
| ENG | SSH2 | SPATA16 | FAM134C | GTF2F2 | KCNJ4 |
| RFPL1 | SNRNP48 | MTSS1L | SFTPB | CENPT | EFNA5 |
| CITED2 | CEACAM5 | CEACAM3 | PLEKHH3 | NT5C | SERPINA3 |
| SAG | ELSPBP1 | CER1 | CHGB | RAB7A | MYH9 |
| NOSTRIN | SCN2B | CHL1 | C9orf142 | ZNF491 | TAC1 |
| TMEM17 | PURG | NIPAL2 | SMC5 | KARS | KCNK4 |
| PIGR | MYH11 | MT3 | PRTN3 | TNPO1 | POLR3E |
| VPS54 | GATM | FBLN2 | SPHK2 | STK40 | KDM5A |
| PTP4A2 | SLC37A4 | SCYL1 | BCAS1 | C2orf82 | TMEM191A |
| MAP9 | CSMD3 | CLGN | MVD | SLC4A2 | SLC24A2 |
| UBAP2 | STMN2 | UBA3 | NUDT1 | TSSC4 | CSNK1E |
| BAG4 | PHYHIP | BTBD1 | MATN4 | VAMP2 | NTM |
| IFNA7 | HHIPL2 | TUBA1C | RPS14 | ZNF587 | PFN1 |
| RHOD | NDUFS2 | LRRC40 | EXOC8 | OR1I1 | KCNA2 |
| LDHA | SNX10 | IPO7 | OPCML | LRP3 | TCF7 |
| GNAQ | PNOC | ABLIM2 | SERPINI1 | WIF1 | PDE1B |
| MYOC | KCMF1 | AGL | ALG2 | GRIN2A | MAPRE3 |
| IFI6 | CDH5 | KPNA5 | CYP11A1 | L1CAM | KLHDC10 |
| RXRB | PSMB8 | HEXIM1 | RILP | HIST1H1A | LPCAT3 |
| INHBE | CENPA | TPI1 | NEUROD1 | GSG2 | SLC15A3 |
| GNL3 | UBE2B | CHRNG | PRCP | OGFRL1 | KIF1A |
| MS4A6E | TRIM56 | IFT20 | SSTR2 | TUBB2A | GRIA3 |
| ZAR1 | RNASE6 | SEMA6C | PLIN5 | NOL6 | CALB1 |
| ERI3 | MDGA2 | LCN10 | NCBP1 | EPHX2 | MTMR1 |
| AACS | GRM6 | PTCRA | MAG | ZC3H3 | ALDH1A1 |
| DPP10 | LIAS | CREG2 | PTER | RHOC | YWHAG |
| GCNT3 | HEBP2 | FAM126A | TRIM9 | CD14 | ABHD8 |
| TDRD9 | TMEM200A | VPS37D | UBB | ABCC1 | VPS13D |
| CCT6A | LMLN | MPV17 | B4GALNT4 | DDX59 | RHOB |
| IL4R | S100A11 | GABRA1 | PLK2 | GPATCH8 | SPG7 |
| GLIPR2 | ZHX1 | EHD1 | CLEC2D | RPA3 | TRHDE |
| PPM1F | SLC44A5 | ZNF219 | ITPKB | HOMER1 | SDF2L1 |
| HSPB6 | CFL2 | NEK7 | MAGED1 | POMGNT1 | AIF1L |
| UAP1L1 | TST | FAM98B | ID3 | CDC42EP3 | ZCCHC2 |
| REV3L | ZNF365 | PDK3 | PPP4C | ZNF787 | FAM131B |
| KCTD3 | FBXL17 | VSNL1 | ATP1B1 | SLIT1 | GPR37L1 |
| AMPH | TBC1D24 | SLC2A10 | LRP5 | BMPR1B | ANKRD33 |
| ZNF417 | GFRA2 | P2RY14 | MRPL13 | ZNF623 | HIF1A |
| PLXNB1 | MAP3K10 | RAB3GAP2 | THRB | FAM168A | CRIP1 |
| RGS11 | ELAVL2 | DGCR6L | NEDD8 | ZNF428 | CHN1 |
| WDR18 | HBB | DDX3Y | MAN2A1 | SYNM | PPP3CA |
| P2RX5 | MAPK4 | CST3 | HLA-DRA | HMGN3 | ASH1L |
| MYL12A | CYP4F11 | PNPLA7 | STXBP1 | GLTSCR2 | DDA1 |
| NME7 | GLIS2 | MAP1B | CNN3 | RASEF | PURB |
| S100A8 | CHRDL1 | EGLN2 | ATP8B1 | CDIPT | PHLDB1 |
| SF3B1 | MED24 | RPL41 | HES4 | AMPD2 | PRDM8 |
| NKX6-2 | ADRA1B | SYT16 | PGF | PSD3 | ALPP |
| ABI2 | PCDH8 | RASGEF1A | SH3BP5 | CCDC125 | EIF4A2 |
| TIMM23 | EPN1 | SLC6A7 | ZNF738 | PRR11 | CCDC106 |
| GJC2 | UBR1 | GPR158 | KCNJ9 | GABRB2 | RTN1 |
| CTXN1 | RANBP3L | TMED8 |  |  |  |
