# Supplementary material for: Identification of tryptophan metabolism-related genes in immunity and immunotherapy in Alzheimer’s disease
Source: Aging (Albany NY). 2023 Nov 20;15(22):13077–99. doi: 10.18632/aging.205220 (PMC10713402; doi:10.18632/aging.205220)
Supplement: Appendix 5 [file aging-15-205220-s006.docx]

**Appendix 5. Co-expression network construction and module detection of clustering.**

**Table 5. Co-expression Network Construction and Module Detection of Clustering.**

| SCP2 | SUSD2 | HES7 | NCBP1 | HP1BP3 | GRID1 |
| --- | --- | --- | --- | --- | --- |
| PDCD7 | GOLT1A | FAM171B | PTER | ITPKA | SH3GLB2 |
| MATN3 | ZNF425 | CLGN | CAMTA2 | SLC25A33 | VPS13D |
| NDUFB3 | DNAH1 | FBF1 | EPHA7 | ZNF430 | AIF1L |
| MST1R | POLE3 | TUBA1C | PCID2 | RHBDL2 | OLA1 |
| CSF3 | PHTF2 | PYROXD1 | PLK2 | FN1 | IFFO1 |
| ELANE | OSBPL6 | E2F5 | DOCK1 | FLOT2 | SH2B2 |
| KCNH4 | CDC20 | SGPP1 | TMED3 | POLE4 | ZCCHC2 |
| DNMBP | ACTG2 | PBX1 | ID3 | NFASC | PLXNB1 |
| IFIH1 | NFE2 | PARP1 | LDLR | SLC16A1 | PPID |
| EFHD2 | GALNT1 | SERPINB9 | ZNF354B | SCO1 | IPO11 |
| FBXO40 | LAPTM4A | GUCY1A2 | ORAI2 | MRPL37 | RASGRF2 |
| GLT1D1 | S100A10 | GDF2 | ZNF649 | CDC27 | MYL12A |
| SS18 | SOX8 | DNAJC16 | MAL2 | CENPB | AGPAT3 |
| SNX31 | ADORA2B | CREG2 | DOCK10 | GOT1 | SMAD1 |
| MCTP1 | HTR2C | FAM126A | PCDHB7 | ARPC1A | SF3B1 |
| STYK1 | PTPMT1 | EYA3 | UBXN7 | SYN3 | FOS |
| NTRK3 | MRFAP1L1 | DTX3L | PAIP1 | SAE1 | PLA2G2D |
| TPSD1 | PNMAL1 | PROK2 | DKK3 | RNASET2 | TRMT11 |
| RFX3 | IMPACT | GABRA1 | EEF1D | DPP6 | ABI2 |
| KCNJ13 | EIF1AX | NKPD1 | TBR1 | RPL11 | PPP4R2 |
| SEC23A | NANOS1 | ZNF613 | C1QC | UBAC1 | WBP2 |
| ANKRD44 | LPIN2 | CNR2 | NEFH | ARHGDIB | ATR |
| ALDH1L1 | DOK5 | ZNF219 | MTUS2 | SEZ6L | ESF1 |
| SYT14 | MGEA5 | LATS1 | IMP3 | GIMAP4 | AMOT |
| CNIH2 | MYH11 | MYEF2 | FNDC3B | ATRX | CACYBP |
| EDEM2 | FGF17 | FSIP1 | ZNF267 | GABARAP | SCNN1D |
| HIST1H4G | VPS4A | P2RY14 | ASCL2 | LNPEP | CHRDL1 |
| CD200 | STMN2 | OXNAD1 | CDC42SE1 | TTYH2 | MEST |
| CCNI | TOP3B | NEFM | TTYH3 | OAF | PCDH8 |
| CDC37L1 | PHYHIP | FLT1 | RASD2 | PKIG | TAF9B |
| ECHDC2 | EVL | TAF11 | PCSK2 | SUMO1 | UBR1 |
| SLC25A2 | PNOC | TIAM1 | AP3B1 | SDHB | TNR |
| CHTF8 | CBX1 | GULP1 | MMP16 | ATP6V0A1 | TDRD1 |
| PPL | C10orf107 | DCTN2 | G0S2 | IFITM3 | MAP1B |
| ETHE1 | CDH5 | S100A12 | RAP1B | GALNT7 | MAP2 |
| MKNK2 | PSMB8 | PCBP1 | SIPA1 | VDAC1 | CACNA2D1 |
| TAF1L | SLC26A11 | BIRC7 | CHRNB1 | PHF6 | SAMHD1 |
| SEMA6D | FEM1C | CCND2 | CRTAC1 | ZNF296 | RPL41 |
| ZBTB6 | ZNF165 | IMMP1L | GABRA4 | KCNA1 | GDA |
| PLA2G2A | TRIM56 | EMP1 | SOX1 | PTPN5 | WASF1 |
| HS3ST3B1 | ZNF76 | KIFAP3 | PHC3 | KIF3A | GPR158 |
| NPY | DNAJA4 | C11orf49 | RICTOR | SYP | DLGAP1 |
| ZMIZ2 | ST6GALNAC6 | HSP90AA1 | PRKAA2 | CACNA1B | LAIR1 |
| FAM65C | IFI44L | AP3S1 | MAPK1IP1L | ARF4 | MFGE8 |
| UCK1 | LIAS | CLSTN1 | ATP5H | SLC25A42 | NUBPL |
| LEF1 | S100A11 | IFITM2 | CAMK4 | CCNL2 | POU3F3 |
| PIK3R2 | KLF9 | HS6ST2 | MED6 | SYNGR3 | THRB |
| CNTFR | SLC44A5 | ALAS1 | FITM2 | PPP2R1A | BZW1 |
| CD74 | LARP1 | ZNF394 | FEM1B | DUSP7 | MAN2A1 |
| SH3PXD2A | PLA2G12A | SRFBP1 | BLOC1S2 | KIRREL3 | HLA-DRA |
| BMP6 | PCYT1A | RAB39B | SLC9A6 | NUMB | PIK3R1 |
| RFPL1 | ZNF365 | RPS11 | FAM84A | PSMC3 | GPR137 |
| GRB14 | TM4SF5 | MAP3K6 | ANK3 | DPYSL3 | CNN3 |
| NOSTRIN | KIAA0430 | PTN | FANCL | THAP5 | INPP5F |
| MAP9 | GFRA2 | CLTB | ITM2B | FZD9 | EZR |
| UBAP2 | SLCO2B1 | SPCS3 | ENO1 | ZDHHC8 | PABPC3 |
| MESP1 | SH3GL3 | ZC3H7B | LYPD3 | HSPB1 | SH3BP5 |
| TNNT3 | FUNDC2 | AKR1C4 | PPP1R15A | SYT1 | ANKRD36B |
| MINK1 | SDHAF2 | YIPF3 | LIME1 | CADM4 | ZNF738 |
| QSER1 | TRIM17 | PLD6 | MTHFD1L | CDKN2AIPNL | PITPNM3 |
| IGSF22 | GLCCI1 | NLGN3 | GTF2F2 | ABAT | PRKACB |
| RXRB | DRD3 | C15orf52 | RAB7A | CLIP3 | KCNJ9 |
| MDM4 | SART1 | NRN1 | ZNF491 | APP | BLZF1 |
| ZAR1 | CX3CR1 | ATXN7 | ACBD5 | NR3C1 | F2R |
| ERI3 | RTN4 | RPRML | PEX3 | ALKBH5 | BTBD2 |
| TIMP4 | SLC22A25 | ADCY3 | SLC4A2 | CUX1 | FBXW12 |
| FAM114A1 | UXS1 | ANXA2P1 | LONRF2 | GPRASP1 | MAP4K2 |
| GABRA5 | CNKSR3 | FAM181B | KIF5A | DCAKD | VDAC2 |
| RARS | FOXO3 | RHO | SLC25A39 | CWF19L1 | FGF13 |
| CCT6A | PCDH7 | USP4 | ZNF587 | B3GAT2 | PRKAR2B |
| CDON | ZNF519 | MSN | MACROD2 | SCN3A | SYNM |
| GLIPR2 | SACS | BRD4 | RPS4X | SERPINA3 | FMR1 |
| HYAL3 | TBC1D5 | SPINT3 | SFN | MYH9 | SAMD12 |
| HSPB6 | B3GNT9 | POLI | ZNF665 | KDM5A | HMGN3 |
| UAP1L1 | DEFB123 | TCEAL4 | NALCN | GJC1 | DNAJC6 |
| RASSF3 | ZNF678 | ZBTB26 | ATF7IP | ZMYND11 | UBE2M |
| NFIC | CBX3 | ZNF506 | EXOC3L2 | SYT2 | ZNF549 |
| ATOX1 | UBE2E3 | DCBLD2 | ANP32E | SCN1B | MORF4L1 |
| AEBP1 | HIBADH | LCAT | GSTT1 | PHGDH | CDC37 |
| GABRD | SDPR | KSR2 | TNKS | MXI1 | DAZAP1 |
| ZNF793 | ZBTB16 | KLHL35 | SLC25A3 | GLIS1 | AMPD2 |
| SFT2D2 | HSPB3 | ZNF180 | RGP1 | ZNF653 | PSD3 |
| CSNK1G2 | NDUFS7 | PLEKHH3 | AIRE | ZNF577 | CCDC125 |
| MAST4 | ITFG2 | BCKDK | ZNF787 | CAPZA1 | CALM1 |
| PTPN1 | HCN1 | CHGB | LY96 | KPNA2 | RNF11 |
| NEFL | CUTA | PRTN3 | ZNF623 | SLC15A3 | GABRB2 |
| F8 | RXFP1 | SQSTM1 | SLC30A3 | ASAH1 | GLUL |
| SPIRE1 | TUSC1 | PIKFYVE | SLITRK2 | CPSF1 | USMG5 |
| TSPYL2 | MAOA | CA2 | CEP97 | C7orf50 | SCN2A |
| NDUFAF2 | KBTBD11 | ZNRF1 | COBLL1 | ADD2 | MALAT1 |
| RHOA | NFAT5 | GLUD1 | BST2 | SGTB | EIF4A2 |
| KY | GNG7 | PDGFRB | CSDE1 | CRIP2 | FRZB |
| ARHGDIA | TECPR2 | RPS14 | GAS2L1 | KIF1A | FBLN2 |
| IL6R | GAS7 | ALG2 | RBPJ | PDCD4 | CD274 |
| CDK7 | CHL1 | RILP | SMCR8 | GRIA3 | MED13 |
| DACT3 | NIPAL2 | NEUROD1 | PEX12 | CALB1 | ALDH1A1 |
| APBB1IP | HLA-DMA | SSTR2 | RPL18 | MTMR1 | C16orf58 |
| HOXC12 | ZFYVE9 | RNF6 | RPL37A |  |  |
