# Supplementary material for: Identification of tryptophan metabolism-related genes in immunity and immunotherapy in Alzheimer’s disease
Source: Aging (Albany NY). 2023 Nov 20;15(22):13077–99. doi: 10.18632/aging.205220 (PMC10713402; doi:10.18632/aging.205220)
Supplement: Appendix 6 [file aging-15-205220-s007.docx]

# Appendix 6. InterGenes.

**Table 6. InterGenes.**

| SCP2 | PCCB | HSP90AA1 | BRAP | UBAC1 | RGS11 |
| --- | --- | --- | --- | --- | --- |
| PDCD7 | FEM1C | AP3S1 | SIPA1 | GTF3C4 | P2RX5 |
| RIMS4 | UBE2B | CLSTN1 | DMAP1 | NACAP1 | TPPP |
| HUWE1 | ZNF76 | AKT3 | PITPNB | ARHGDIB | FOXO4 |
| GIT2 | TTC38 | ZNF394 | CHRNB1 | SEZ6L | IPO11 |
| DNMBP | DNAJA4 | SIRT3 | CRTAC1 | GIMAP4 | RASGRF2 |
| IFIH1 | ST6GALNAC6 | FAM3A | GRIN2C | ATRX | MYL12A |
| EFHD2 | LMLN | LIMD1 | ACVR1B | GABARAP | AGPAT3 |
| GLT1D1 | KLF9 | GAL3ST3 | STOML2 | LNPEP | NEK9 |
| WIPI2 | ZHX1 | RGL4 | SOX1 | TTYH2 | SMAD1 |
| SS18 | SLC44A5 | RAB39B | TCTN2 | ZNF710 | PPIB |
| FRS2 | LARP1 | MAP3K6 | DAGLB | OAF | KCNQ5 |
| LPGAT1 | CFL2 | PTN | PHC3 | PKIG | TEAD1 |
| INPP4A | PLA2G12A | P4HA1 | RICTOR | SUMO1 | SF3B1 |
| MCTP1 | PCYT1A | DNAJB11 | FGD4 | DOPEY2 | PLA2G2D |
| KIF7 | SOD2 | SPCS3 | PRKAA2 | KLC2 | TRMT11 |
| WFS1 | PJA1 | ZC3H7B | VOPP1 | BSN | COX16 |
| NTRK3 | FBXL17 | YIPF3 | GPN3 | SDHB | GOLGA7 |
| LSM3 | KIAA0430 | GDAP1 | MAPK1IP1L | PDZD11 | ABI2 |
| RFX3 | TBC1D24 | LRRC52 | SYTL4 | ITPR3 | PI4KA |
| LMO3 | FOXG1 | WBSCR17 | KCNAB2 | SEC61A1 | ANKH |
| SEC23A | SLCO2B1 | C4orf33 | ATP5H | GALNT7 | HAVCR2 |
| SON | SCN5A | ZNF528 | CAMK4 | VDAC1 | TIMM23 |
| ANKRD44 | ZNF557 | PLD6 | FGF14 | RRAGB | PPP4R2 |
| PHF1 | SH3GL3 | FAM106A | PLEKHA4 | PHF6 | WBP2 |
| SLC25A25 | FUNDC2 | NLGN3 | MED6 | ZNF296 | ATR |
| LTA | VAPA | ZBTB48 | CNKSR2 | DALRD3 | ESF1 |
| CCNI | SDHAF2 | ATXN7 | COPA | ALDOA | GJC2 |
| CDC37L1 | MADCAM1 | MRPL38 | STARD4 | PTPN5 | ZNF445 |
| ZFAND5 | DRD3 | KLHL22 | FSCN1 | KIF3A | CCDC106 |
| ECHDC2 | SART1 | DAB2 | FITM2 | DRD4 | AMOT |
| CHTF8 | ZNF480 | ADCY3 | FEM1B | CACNA1B | CHN1 |
| BRMS1L | SETD7 | ANXA2P1 | BLOC1S2 | SYAP1 | RPLP0 |
| NPAS1 | RTN4 | FAM181B | SLC9A6 | GPR27 | ELAVL2 |
| PATZ1 | MAP6D1 | RASGRP3 | ARL6 | SLC17A5 | CACYBP |
| RCC1 | ANKRD13D | RHO | FAM84A | BCO2 | SCNN1D |
| CA4 | SLC22A25 | MYO18B | SNAPC3 | ARF4 | MAPK4 |
| MKNK2 | LBR | PTGR2 | HGS | AHRR | CYP4F11 |
| DBNDD1 | PDXK | GATC | ANK3 | RFK | GLIS2 |
| SEMA6D | UXS1 | PLCB3 | DNAJC1 | EVI5L | CHRDL1 |
| HS3ST3B1 | CNKSR3 | USP4 | FANCL | CTBP2 | MEST |
| ZMIZ2 | FES | CYP1A2 | ISG20 | SLC25A42 | ADRA1B |
| RBM19 | INTS3 | MSN | FXYD5 | HCP5 | MCM8 |
| AAK1 | FOXO3 | BRD4 | MFAP4 | TRABD | PCDH8 |
| NDUFS3 | PCDH7 | SPINT3 | ITM2B | FAM19A5 | TAF9B |
| MX1 | GTPBP10 | POLI | ENO1 | CCNL2 | EPN1 |
| GIN1 | SLC12A9 | FAM134A | XPOT | ZNF48 | UBR1 |
| PIGQ | SACS | RPL14 | LYPD3 | SYNGR3 | RTN1 |
| SNTB1 | COPS7A | TCEAL4 | PPP1R15A | HIPK3 | PPP3CA |
| UCK1 | TBC1D5 | ZBTB26 | LIME1 | PPP2R1A | ASH1L |
| CCDC82 | B3GNT9 | ZNF506 | ATP13A3 | MEIS2 | CHRNA5 |
| STK4 | TEKT1 | SERINC5 | MTHFD1L | CACNG8 | AKR1C1 |
| SLC19A3 | GTF3C6 | DCBLD2 | GTF2F2 | DUSP7 | DGCR6L |
| PIK3R2 | ZNF678 | LCAT | NT5C | DDX51 | TNR |
| PLEKHB2 | CBX3 | KSR2 | CHST7 | KIRREL3 | TDRD1 |
| PRKCH | UBE2E3 | KLHL35 | RHBDF1 | PLEKHM2 | CST3 |
| SFT2D1 | NCSTN | ZNF180 | LLPH | NUMB | PNPLA7 |
| AAGAB | AMPD3 | SFTPB | RAB7A | PSMC3 | MAP1B |
| SH3PXD2A | GALC | SMUG1 | ZNF491 | TXLNA | EGLN2 |
| BMP6 | HIBADH | PLEKHH3 | KARS | SCD | ITSN2 |
| AMOTL2 | B9D2 | BCKDK | ACBD5 | PANX2 | MAP2 |
| ZNF253 | RGMA | C9orf142 | RHBDL3 | DPYSL3 | CACNA2D1 |
| UBAP2 | SNX9 | MACF1 | GPT2 | THAP5 | SAMHD1 |
| MESP1 | HCFC1R1 | FAM120B | STK40 | FZD9 | MTCP1 |
| TNNT3 | KIAA1683 | SMC5 | PEX3 | ZDHHC8 | SYT16 |
| BAG4 | DCAF7 | SATB1 | LONRF2 | HSPB1 | RASGEF1A |
| MINK1 | ZBTB16 | FOXP1 | KIF5A | KCNJ3 | GDA |
| QSER1 | DNALI1 | SPHK2 | SLC25A39 | CADM4 | WASF1 |
| TTC23 | VPS13C | SQSTM1 | SNX18 | RFTN2 | GPR158 |
| C11orf58 | TRPM3 | ASB1 | ZNF587 | FBXO9 | TMEM156 |
| GNAQ | NDUFS7 | TAF6 | APBB2 | CLDND2 | DLGAP1 |
| RXRB | TMEM161A | ZNF420 | LRP3 | ANK2 | TMED8 |
| C1orf43 | HCN1 | PIKFYVE | USP20 | STIP1 | LAIR1 |
| MDM4 | CUTA | FAM188A | MACROD2 | CDKN2AIPNL | MFGE8 |
| OXER1 | IGFBP5 | MVD | L1CAM | ABAT | NUBPL |
| RARS | OSBP | ZNRF1 | GSG2 | CLIP3 | DDA1 |
| CCT6A | TUSC1 | GLUD1 | OGFRL1 | APP | DVL1 |
| CDON | MAOA | PDGFRB | TUBB2A | BEX1 | POU3F3 |
| GLIPR2 | KBTBD11 | EXOC8 | TRAPPC10 | NR3C1 | SLC12A7 |
| REV3L | ZSCAN29 | KIF27 | NOL6 | ALKBH5 | PURB |
| KCTD3 | SLC35D1 | ALG2 | ZNF665 | ASCC2 | THRB |
| NFIC | TMEM219 | RILP | ZC3H3 | CUX1 | HS6ST3 |
| UBQLNL | COPS2 | PRR18 | NALCN | GPRASP1 | BZW1 |
| ATOX1 | RABGGTB | PRCP | RHOC | DCAKD | MAN2A1 |
| ZNF793 | CLEC14A | SSTR2 | ATF7IP | ZNF121 | NOTCH2 |
| ELF1 | SUPT7L | RNF6 | EXOC3L2 | CWF19L1 | HLA-DRA |
| WDR59 | NFAT5 | PLIN5 | ANP32E | B3GAT2 | KCNC1 |
| SFT2D2 | GNG7 | SDAD1 | ABCC1 | DUSP3 | PNCK |
| APEH | NEU3 | CD274 | DDX59 | ASGR1 | PIK3R1 |
| KLK3 | DHDH | NCBP1 | TNKS | ALOX5AP | GPR137 |
| RNF31 | TECPR2 | CIB1 | GPATCH8 | SCN3A | SLC5A8 |
| CSNK1G2 | GAS7 | CAMTA2 | SLC25A3 | EFNA5 | STXBP1 |
| MAST4 | CHL1 | EPHA7 | USP31 | PPIG | ADCYAP1R1 |
| HPS6 | VASN | TRIM9 | POMGNT1 | CAMKK1 | CNN3 |
| PTPN1 | NIPAL2 | UBB | PRPF40A | SYT11 | ATP8B1 |
| MGLL | C2orf88 | TMEM65 | RGP1 | MYH9 | INPP5F |
| PPP1R3C | HLA-DMA | B4GALNT4 | AIRE | ATP8A1 | HES4 |
| SEC11C | JMJD8 | PCID2 | ZNF787 | NFIX | EZR |
| F8 | ZFYVE9 | RNGTT | LY96 | MFSD10 | LILRB3 |
| SPIRE1 | SCYL1 | PPM1L | BMPR1B | POLR3E | PABPC3 |
| TSPYL2 | FAM171B | DOCK1 | CRYM | KDM5A | PGF |
| ATG16L1 | ASAP3 | SPC24 | ZNF623 | GJC1 | JAKMIP1 |
| TSNAX | FBF1 | TMED3 | SLITRK2 | NCAM1 | SH3BP5 |
| RHOA | UBA3 | MAGED1 | NPTN | SLC24A2 | ANKRD36B |
| SFXN5 | BTBD1 | GRM8 | CEP97 | ZMYND11 | ZNF738 |
| ARHGEF10 | IRF9 | CCND3 | NEK5 | SYT2 | PITPNM3 |
| KY | LRRC40 | SLC9A1 | IARS | SCN1B | PRKACB |
| ARHGDIA | IPO7 | ATP1B1 | COBLL1 | PHGDH | LILRB1 |
| IL6R | AGL | LRP5 | LMNB2 | MXI1 | KCNJ9 |
| CACNA1H | PYROXD1 | LDLR | TMEM145 | HDLBP | NLRP8 |
| DACT3 | RGS18 | ZNF354B | ZDHHC1 | PFN1 | PPIA |
| APBB1IP | IFT20 | PSMB10 | FCAR | KCNA2 | BLZF1 |
| WDR3 | SGPP1 | PFDN2 | TMTC3 | ZNF786 | MAGT1 |
| CFB | LHFPL3 | NEUROG2 | CSDE1 | TCF7 | PHLDB1 |
| RFWD3 | PBX1 | ORAI2 | ING5 | GLIS1 | F2R |
| FRZB | PARP1 | ZNF649 | GAS2L1 | ZNF653 | PRDM8 |
| SUSD2 | TNFRSF10C | DOCK10 | RBPJ | CLCN7 | ERBB4 |
| LCORL | SERPINB9 | PCDHB7 | SMCR8 | MAPRE3 | BTBD2 |
| SH2D1A | GUCY1A2 | ROBO1 | PEX12 | KLHDC10 | FBXW12 |
| NDUFS4 | RAVER2 | DUT | LHPP | ZNF577 | MAP4K2 |
| ZNF425 | DNAJC16 | TIMM17A | SLC44A2 | CAPZA1 | VDAC2 |
| NDUFB1 | EYA3 | ATP6AP1 | SLC6A13 | KPNA2 | FGF13 |
| DNAH1 | SAMD14 | METAP2 | BAIAP2L2 | PRIMA1 | PRKAR2B |
| OR3A3 | CCDC102A | UBXN7 | RPL18 | SLC15A3 | FAM168A |
| POLE3 | NKPD1 | SPRED1 | RPL37A | ASAH1 | ZNF428 |
| PHTF2 | ZNF613 | PAIP1 | MED13 | CPSF1 | SYNM |
| RASA1 | EHD1 | ANP32A | PRKAR2A | KCNH6 | FMR1 |
| OSBPL6 | CNR2 | CD81 | HP1BP3 | C7orf50 | SAMD12 |
| EPC1 | ZNF219 | NCOA2 | ITPKA | ADD2 | HMGN3 |
| NFE2 | NEK7 | RCOR1 | FGFR1 | SGTB | GLTSCR2 |
| GALNT1 | LATS1 | EEF1D | PAFAH1B2 | CRIP2 | DNAJC6 |
| LAPTM4A | RBM33 | TRIM37 | FARSB | KIF1A | UBE2M |
| ACO1 | MYEF2 | C1orf162 | POU2F2 | PDCD4 | ZNF549 |
| TRRAP | FAM98B | NPEPPS | SLC25A33 | GRIA3 | SHCBP1 |
| GPATCH1 | PPM1M | MTUS2 | ZNF430 | MTMR1 | MORF4L1 |
| GATS | RPN1 | GRAMD1B | RHBDL2 | C16orf58 | CDC37 |
| PTPMT1 | DMXL1 | IMP3 | FN1 | ALDH1A1 | DAZAP1 |
| MRFAP1L1 | ARMCX6 | NLRC5 | FLOT2 | GRID1 | RPL8 |
| FOXN3 | HNRNPUL1 | FNDC3B | POLE4 | CDC42 | CDIPT |
| TACC1 | PNPLA3 | MAFK | NFASC | YWHAG | PRKCZ |
| IMPACT | DUSP23 | NENF | SLC16A1 | SH3GLB2 | AMPD2 |
| EIF1AX | KCNK12 | ZNF267 | SNRNP40 | VPS13D | SLC41A3 |
| NANOS1 | FAIM2 | QKI | GNAS | RHOB | DTWD2 |
| FIGN | NPY1R | PDE6C | GPX8 | RNF139 | PSD3 |
| LPIN2 | H2AFV | ASCL2 | EML3 | SPG7 | CCDC125 |
| SSH2 | HK1 | CROCC | TRPM4 | AIF1L | C8orf37 |
| FAM117A | TAF11 | ADAM33 | SCO1 | ZNF862 | CCBE1 |
| SNRNP48 | TIAM1 | PATE2 | MRPL37 | ALPL | CALM1 |
| PPP1R11 | SLC27A3 | SPG21 | MFSD3 | FKBP9 | PRR11 |
| DOK5 | DCTN2 | CDC42SE1 | DDX42 | DOCK7 | RNF11 |
| MGEA5 | KLF10 | MEF2A | CDC27 | OLA1 | GABRB2 |
| TMEM120B | PCBP1 | TTYH3 | CAMK2D | IFFO1 | LMOD3 |
| VPS4A | EIF2AK4 | RASD2 | CENPB | EMILIN3 | GLUL |
| SLC37A4 | ZNF703 | CNTN4 | POLR2A | SH2B2 | CTXN1 |
| CSMD3 | IFT80 | PFN2 | CYFIP1 | ZCCHC2 | DUSP19 |
| DUS4L | ACSS1 | NUDT14 | ARPC1A | SLC7A11 | USMG5 |
| TOP3B | PRLH | RANBP9 | TOP1 | USP5 | SCN2A |
| EVL | GAK | AP3B1 | SYN3 | FAM131B | NPM1 |
| NDUFS2 | PRDM11 | MMP16 | DOCK2 | HCFC1 | QRFPR |
| NKIRAS2 | CCND2 | G0S2 | ATCAY | GPR37L1 | MALAT1 |
| AFF3 | IMMP1L | PLAG1 | SAE1 | TNFSF14 | DMC1 |
| CBX1 | LRRTM2 | RAP1B | RNASET2 | AGAP1 | ALPP |
| KCMF1 | FAM35A | CECR2 | DPP6 | PLXNB1 | EIF4A2 |
| SLC26A11 | C11orf49 | ENTPD4 | RPL11 | PPID |  |
