# Supplementary material for: Identification of tryptophan metabolism-related genes in immunity and immunotherapy in Alzheimer’s disease
Source: Aging (Albany NY). 2023 Nov 20;15(22):13077–99. doi: 10.18632/aging.205220 (PMC10713402; doi:10.18632/aging.205220)
Supplement: Appendix 7 [file aging-15-205220-s008.docx]

# Appendix 7. Important genes were predicted based on the eXtreme gradient boosting (XGB) model.

**Table 7. ImportanceGene.XGB.**

| variable | permutation | dropout_loss | label |
| --- | --- | --- | --- |
| PCCB | 0 | 0.313111132 | XGB |
| TEAD1 | 0 | 0.314946201 | XGB |
| FARSB | 0 | 0.316038415 | XGB |
| NFASC | 0 | 0.318069793 | XGB |
| EZR | 0 | 0.324218762 | XGB |
