# Supplementary material for: Identification of tryptophan metabolism-related genes in immunity and immunotherapy in Alzheimer’s disease
Source: Aging (Albany NY). 2023 Nov 20;15(22):13077–99. doi: 10.18632/aging.205220 (PMC10713402; doi:10.18632/aging.205220)
Supplement: Appendix 8 [file aging-15-205220-s009.docx]

# Appendix 8. The correlation analysis between the five hub genes and age.

**Table 6. corResult.**

| Gene | Clinical | cor | pvalue |
| --- | --- | --- | --- |
| PCCB | Age | -0.198380048 | 0.013343634 |
| TEAD1 | Age | 0.059805799 | 0.459778785 |
| FARSB | Age | -0.117815384 | 0.14428831 |
| NFASC | Age | 0.03848909 | 0.634442932 |
| EZR | Age | - | - |
