# Supplementary material for: Identification of tryptophan metabolism-related genes in immunity and immunotherapy in Alzheimer’s disease
Source: Aging (Albany NY). 2023 Nov 20;15(22):13077–99. doi: 10.18632/aging.205220 (PMC10713402; doi:10.18632/aging.205220)
Supplement: Appendix 9 [file aging-15-205220-s010.docx]

# Appendix 9. Drug-gene interactions of all intergenes.

Table 9. Drug-gene interactions of all intergenes

| Search_term | Match_term | Gene | Drug | Interaction_types | Sources |
| --- | --- | --- | --- | --- | --- |
| NTRK3 | NTRK3 | NTRK3 | LAROTRECTINIB | inhibitor | ChemblInteractions\|CIViC\|TTD\|OncoKB |
| NTRK3 | NTRK3 | NTRK3 | SB-202190 | Unknown | DTC |
| NTRK3 | NTRK3 | NTRK3 | ILORASERTIB | Unknown | DTC |
| NTRK3 | NTRK3 | NTRK3 | SNS-314 | Unknown | DTC |
| NTRK3 | NTRK3 | NTRK3 | GW441756X | Unknown | DTC |
| NTRK3 | NTRK3 | NTRK3 | ALTIRATINIB | inhibitor | ChemblInteractions\|TTD |
| NTRK3 | NTRK3 | NTRK3 | MLN-8054 | Unknown | DTC |
| NTRK3 | NTRK3 | NTRK3 | MK-2461 | Unknown | TTD |
| NTRK3 | NTRK3 | NTRK3 | CYC-116 | Unknown | DTC |
| NTRK3 | NTRK3 | NTRK3 | LINIFANIB | Unknown | DTC |
| NTRK3 | NTRK3 | NTRK3 | TANDUTINIB | Unknown | DTC |
| NTRK3 | NTRK3 | NTRK3 | THYROXINE | Unknown | NCI |
| NTRK3 | NTRK3 | NTRK3 | GW632580X | Unknown | DTC |
| NTRK3 | NTRK3 | NTRK3 | GAMBOGIC AMIDE | Unknown | DTC |
| NTRK3 | NTRK3 | NTRK3 | AZD-7451 | inhibitor | ChemblInteractions |
| NTRK3 | NTRK3 | NTRK3 | TOZASERTIB | Unknown | DTC |
| NTRK3 | NTRK3 | NTRK3 | HESPERADIN | inhibitor | DTC |
| NTRK3 | NTRK3 | NTRK3 | LESTAURTINIB | inhibitor | TdgClinicalTrial\|ChemblInteractions |
| NTRK3 | NTRK3 | NTRK3 | ENTRECTINIB | inhibitor | DTC\|CIViC\|PharmGKB\|OncoKB |
| NTRK3 | NTRK3 | NTRK3 | PD-0166285 | Unknown | DTC |
| NTRK3 | NTRK3 | NTRK3 | DORAMAPIMOD | Unknown | DTC |
| NTRK3 | NTRK3 | NTRK3 | SORAFENIB | Unknown | DTC |
| NTRK3 | NTRK3 | NTRK3 | CEP-2563 | inhibitor | ChemblInteractions |
| NTRK3 | NTRK3 | NTRK3 | PLX-7486 | inhibitor | ChemblInteractions |
| NTRK3 | NTRK3 | NTRK3 | R-406 | Unknown | DTC |
| NTRK3 | NTRK3 | NTRK3 | ALISERTIB | Unknown | DTC |
| NTRK3 | NTRK3 | NTRK3 | DOVITINIB | Unknown | DTC |
| NTRK3 | NTRK3 | NTRK3 | RG-1530 | Unknown | DTC |
| NTRK3 | NTRK3 | NTRK3 | AST-487 | Unknown | DTC |
| NTRK3 | NTRK3 | NTRK3 | AZD-6918 | inhibitor | ChemblInteractions |
| NTRK3 | NTRK3 | NTRK3 | SP-600125 | Unknown | DTC |
| NTRK3 | NTRK3 | NTRK3 | REPOTRECTINIB | inhibitor | CIViC |
| LTA | LTA | LTA | CARBAMAZEPINE | Unknown | PharmGKB |
| LTA | LTA | LTA | BAMINERCEPT | inhibitor | TdgClinicalTrial\|ChemblInteractions |
| LTA | LTA | LTA | PATECLIZUMAB | inhibitor | ChemblInteractions |
| LTA | LTA | LTA | ABACAVIR | Unknown | PharmGKB |
| CA4 | CA4 | CA4 | SULFAMYLON | Unknown | TTD |
| CA4 | CA4 | CA4 | METHAZOLAMIDE | inhibitor | ChemblInteractions |
| CA4 | CA4 | CA4 | ETHOXZOLAMIDE | inhibitor | ChemblInteractions |
| CA4 | CA4 | CA4 | PARABEN | Unknown | TTD |
| CA4 | CA4 | CA4 | PHENOL | Unknown | TTD |
| CA4 | CA4 | CA4 | TOPIRAMATE | inhibitor | TdgClinicalTrial\|ChemblInteractions\|TEND |
| CA4 | CA4 | CA4 | ACETAZOLAMIDE | inhibitor | ChemblInteractions |
| CA4 | CA4 | CA4 | CHLOROTHIAZIDE | inhibitor | TdgClinicalTrial\|TEND |
| CA4 | CA4 | CA4 | TRICHLORMETHIAZIDE | inhibitor | TdgClinicalTrial\|TEND |
| CA4 | CA4 | CA4 | DICHLORPHENAMIDE | inhibitor | ChemblInteractions |
| CA4 | CA4 | CA4 | ACETAZOLAMIDE SODIUM | inhibitor | ChemblInteractions |
| CA4 | CA4 | CA4 | BRINZOLAMIDE | inhibitor | TTD |
| CA4 | CA4 | CA4 | SULFAMIDE | Unknown | TTD |
| CA4 | CA4 | CA4 | CURCUMIN | Unknown | TTD |
| MKNK2 | MKNK2 | MKNK2 | SORAFENIB | Unknown | DTC |
| MKNK2 | MKNK2 | MKNK2 | GEFITINIB | Unknown | DTC |
| MKNK2 | MKNK2 | MKNK2 | TOMIVOSERTIB | inhibitor | TTD |
| MKNK2 | MKNK2 | MKNK2 | ERLOTINIB | Unknown | DTC |
| MKNK2 | MKNK2 | MKNK2 | SNS-314 | Unknown | DTC |
| MKNK2 | MKNK2 | MKNK2 | LINIFANIB | Unknown | DTC |
| MKNK2 | MKNK2 | MKNK2 | R-406 | Unknown | DTC |
| MKNK2 | MKNK2 | MKNK2 | CENISERTIB | Unknown | DTC |
| MKNK2 | MKNK2 | MKNK2 | ILORASERTIB | Unknown | DTC |
| MKNK2 | MKNK2 | MKNK2 | CYC-116 | Unknown | DTC |
| MKNK2 | MKNK2 | MKNK2 | TAE-684 | Unknown | DTC |
| MKNK2 | MKNK2 | MKNK2 | RG-1530 | Unknown | DTC |
| MKNK2 | MKNK2 | MKNK2 | GSK-269962A | Unknown | DTC |
| MKNK2 | MKNK2 | MKNK2 | CHEMBL225519 | Unknown | DTC |
| MKNK2 | MKNK2 | MKNK2 | CHEMBL541400 | Unknown | DTC |
| MKNK2 | MKNK2 | MKNK2 | SP-600125 | Unknown | DTC |
| NDUFS3 | NDUFS3 | NDUFS3 | NV-128 | inhibitor | ChemblInteractions |
| NDUFS3 | NDUFS3 | NDUFS3 | METFORMIN HYDROCHLORIDE | inhibitor | ChemblInteractions |
| NDUFS3 | NDUFS3 | NDUFS3 | ME-344 | inhibitor | ChemblInteractions |
| STK4 | STK4 | STK4 | HESPERADIN | inhibitor | DTC |
| SLC19A3 | SLC19A3 | SLC19A3 | METFORMIN | Unknown | PharmGKB |
| PIK3R2 | PIK3R2 | PIK3R2 | PF-04691502 | inhibitor | MyCancerGenome\|ChemblInteractions |
| PIK3R2 | PIK3R2 | PIK3R2 | OMIPALISIB | inhibitor | ChemblInteractions\|MyCancerGenomeClinicalTrial |
| PIK3R2 | PIK3R2 | PIK3R2 | CYCLOPHOSPHAMIDE | Unknown | PharmGKB |
| PIK3R2 | PIK3R2 | PIK3R2 | VEMURAFENIB | Unknown | CIViC |
| PIK3R2 | PIK3R2 | PIK3R2 | GEDATOLISIB | inhibitor | MyCancerGenome\|ChemblInteractions\|MyCancerGenomeClinicalTrial |
| PIK3R2 | PIK3R2 | PIK3R2 | BGT-226 | inhibitor | ChemblInteractions |
| PIK3R2 | PIK3R2 | PIK3R2 | SAMOTOLISIB | inhibitor | ChemblInteractions |
| PIK3R2 | PIK3R2 | PIK3R2 | BUPARLISIB | inhibitor | MyCancerGenome\|ChemblInteractions |
| PIK3R2 | PIK3R2 | PIK3R2 | DACTOLISIB | inhibitor | MyCancerGenome\|ChemblInteractions\|MyCancerGenomeClinicalTrial |
| PIK3R2 | PIK3R2 | PIK3R2 | ZSTK-474 | inhibitor | ChemblInteractions |
| PIK3R2 | PIK3R2 | PIK3R2 | TASELISIB | inhibitor | ChemblInteractions |
| PIK3R2 | PIK3R2 | PIK3R2 | PWT-33587 | inhibitor | ChemblInteractions |
| PIK3R2 | PIK3R2 | PIK3R2 | AZD-6482 | inhibitor | ChemblInteractions |
| PIK3R2 | PIK3R2 | PIK3R2 | WX-037 | inhibitor | ChemblInteractions |
| PIK3R2 | PIK3R2 | PIK3R2 | EPIRUBICIN | Unknown | PharmGKB |
| PIK3R2 | PIK3R2 | PIK3R2 | SONOLISIB | inhibitor | MyCancerGenome\|ChemblInteractions\|MyCancerGenomeClinicalTrial |
| PIK3R2 | PIK3R2 | PIK3R2 | APITOLISIB | inhibitor | MyCancerGenome\|ChemblInteractions |
| PIK3R2 | PIK3R2 | PIK3R2 | PI-103 | inhibitor | MyCancerGenome |
| PIK3R2 | PIK3R2 | PIK3R2 | PILARALISIB | inhibitor | MyCancerGenome\|ChemblInteractions |
| PIK3R2 | PIK3R2 | PIK3R2 | COPANLISIB | inhibitor | MyCancerGenome\|ChemblInteractions\|MyCancerGenomeClinicalTrial |
| PIK3R2 | PIK3R2 | PIK3R2 | VOXTALISIB | inhibitor | MyCancerGenome\|ChemblInteractions |
| PIK3R2 | PIK3R2 | PIK3R2 | RG-7666 | inhibitor | ChemblInteractions |
| PIK3R2 | PIK3R2 | PIK3R2 | GSK-1059615 | inhibitor | ChemblInteractions |
| PIK3R2 | PIK3R2 | PIK3R2 | PICTILISIB | inhibitor | MyCancerGenome\|ChemblInteractions |
| PIK3R2 | PIK3R2 | PIK3R2 | ALPELISIB | inhibitor | MyCancerGenome\|MyCancerGenomeClinicalTrial |
| PIK3R2 | PIK3R2 | PIK3R2 | QUERCETIN | inhibitor | MyCancerGenome |
| PIK3R2 | PIK3R2 | PIK3R2 | DS-7423 | inhibitor | ChemblInteractions |
| PIK3R2 | PIK3R2 | PIK3R2 | FLUOROURACIL | Unknown | PharmGKB |
| PIK3R2 | PIK3R2 | PIK3R2 | PANULISIB | inhibitor | ChemblInteractions |
| PIK3R2 | PIK3R2 | PIK3R2 | PA-799 | inhibitor | ChemblInteractions |
| PIK3R2 | PIK3R2 | PIK3R2 | PUQUITINIB | inhibitor | ChemblInteractions |
| PIK3R2 | PIK3R2 | PIK3R2 | INFIGRATINIB | inhibitor | MyCancerGenome |
| PIK3R2 | PIK3R2 | PIK3R2 | SF-1126 | inhibitor | ChemblInteractions |
| PIK3R2 | PIK3R2 | PIK3R2 | VS-5584 | inhibitor | ChemblInteractions |
| PIK3R2 | PIK3R2 | PIK3R2 | GSK-2636771 | inhibitor | MyCancerGenome |
| PIK3R2 | PIK3R2 | PIK3R2 | RECILISIB | inhibitor | ChemblInteractions |
| PRKCH | PRKCH | PRKCH | CEP-2563 | inhibitor | ChemblInteractions |
| PRKCH | PRKCH | PRKCH | BRYOSTATIN | Unknown | TdgClinicalTrial |
| PRKCH | PRKCH | PRKCH | GSK-690693 | inhibitor | ChemblInteractions |
| PRKCH | PRKCH | PRKCH | SOTRASTAURIN | inhibitor | ChemblInteractions |
| PRKCH | PRKCH | PRKCH | MIDOSTAURIN | inhibitor | ChemblInteractions |
| PRKCH | PRKCH | PRKCH | QUERCETIN | inhibitor | MyCancerGenome |
| PRKCH | PRKCH | PRKCH | UCN-01 | inhibitor | ChemblInteractions |
| TNNT3 | TNNT3 | TNNT3 | TIRASEMTIV | activator | ChemblInteractions |
| MINK1 | MINK1 | MINK1 | CHEMBL541400 | Unknown | DTC |
| MINK1 | MINK1 | MINK1 | PD-0166285 | Unknown | DTC |
| MINK1 | MINK1 | MINK1 | SB-242235 | Unknown | DTC |
| MINK1 | MINK1 | MINK1 | ILORASERTIB | Unknown | DTC |
| MINK1 | MINK1 | MINK1 | ERLOTINIB | Unknown | DTC |
| MINK1 | MINK1 | MINK1 | CEDIRANIB | Unknown | DTC |
| MINK1 | MINK1 | MINK1 | DOVITINIB | Unknown | DTC |
| MINK1 | MINK1 | MINK1 | PF-00562271 | Unknown | DTC |
| MINK1 | MINK1 | MINK1 | SNS-314 | Unknown | DTC |
| MINK1 | MINK1 | MINK1 | TOZASERTIB | Unknown | DTC |
| MINK1 | MINK1 | MINK1 | AZD-1080 | Unknown | DTC |
| MINK1 | MINK1 | MINK1 | DASATINIB | Unknown | DTC |
| MINK1 | MINK1 | MINK1 | SORAFENIB | Unknown | DTC |
| MINK1 | MINK1 | MINK1 | SB-220025 | Unknown | DTC |
| MINK1 | MINK1 | MINK1 | LINIFANIB | Unknown | DTC |
| MINK1 | MINK1 | MINK1 | SOTRASTAURIN | Unknown | DTC |
| MINK1 | MINK1 | MINK1 | OSI-632 | Unknown | DTC |
| MINK1 | MINK1 | MINK1 | CHEMBL379975 | Unknown | DTC |
| MINK1 | MINK1 | MINK1 | ALSTERPAULLONE | Unknown | DTC |
| MINK1 | MINK1 | MINK1 | CHEMBL225519 | Unknown | DTC |
| MINK1 | MINK1 | MINK1 | TAE-684 | Unknown | DTC |
| MINK1 | MINK1 | MINK1 | CENISERTIB | Unknown | DTC |
| MINK1 | MINK1 | MINK1 | GW441756X | Unknown | DTC |
| MINK1 | MINK1 | MINK1 | TAK-715 | Unknown | DTC |
| MINK1 | MINK1 | MINK1 | GEFITINIB | Unknown | DTC |
| MINK1 | MINK1 | MINK1 | SP-600125 | Unknown | DTC |
| MINK1 | MINK1 | MINK1 | SB-203580 | Unknown | DTC |
| GNAQ | GNAQ | GNAQ | VERTEPORFIN | Unknown | JAX-CKB |
| GNAQ | GNAQ | GNAQ | EVEROLIMUS | Unknown | JAX-CKB |
| GNAQ | GNAQ | GNAQ | SELUMETINIB | Unknown | JAX-CKB\|DoCM |
| GNAQ | GNAQ | GNAQ | CGM-097 | Unknown | JAX-CKB |
| GNAQ | GNAQ | GNAQ | BINIMETINIB | Unknown | JAX-CKB |
| GNAQ | GNAQ | GNAQ | REFAMETINIB | Unknown | JAX-CKB |
| GNAQ | GNAQ | GNAQ | ENZASTAURIN | Unknown | JAX-CKB |
| GNAQ | GNAQ | GNAQ | PLX-4720 | Unknown | CIViC |
| GNAQ | GNAQ | GNAQ | OMIPALISIB | Unknown | JAX-CKB |
| GNAQ | GNAQ | GNAQ | SOTRASTAURIN | Unknown | JAX-CKB |
| GNAQ | GNAQ | GNAQ | TRAMETINIB | Unknown | JAX-CKB |
| GNAQ | GNAQ | GNAQ | TAK-733 | Unknown | JAX-CKB |
| GNAQ | GNAQ | GNAQ | PD-0325901 | Unknown | JAX-CKB |
| RXRB | RXRB | RXRB | TAZAROTENE | agonist | TdgClinicalTrial\|TEND |
| RXRB | RXRB | RXRB | TRETINOIN | agonist | TdgClinicalTrial\|TEND |
| RXRB | RXRB | RXRB | ACITRETIN | agonist | ChemblInteractions |
| RXRB | RXRB | RXRB | MOFAROTENE | modulator | ChemblInteractions |
| RXRB | RXRB | RXRB | BEXAROTENE | activator\|agonist | TALC\|TdgClinicalTrial\|ChemblInteractions\|TEND |
| RXRB | RXRB | RXRB | ALITRETINOIN | agonist | ChemblInteractions\|TEND\|TTD |
| RXRB | RXRB | RXRB | ETRETINATE | agonist | ChemblInteractions |
| RXRB | RXRB | RXRB | ADAPALENE | agonist | TdgClinicalTrial\|TEND |
| RXRB | RXRB | RXRB | IRX-4204 | agonist | ChemblInteractions |
| MDM4 | MDM4 | MDM4 | DOCETAXEL | Unknown | PharmGKB |
| MDM4 | MDM4 | MDM4 | EPIRUBICIN | Unknown | PharmGKB |
| MDM4 | MDM4 | MDM4 | ATEZOLIZUMAB | Unknown | CIViC |
| MDM4 | MDM4 | MDM4 | PEMBROLIZUMAB | Unknown | CIViC |
| MDM4 | MDM4 | MDM4 | NIVOLUMAB | Unknown | CIViC |
| OXER1 | OXER1 | OXER1 | 5-OXO-ETE | agonist | DTC |
| RARS | RARS1 | RARS1 | CISPLATIN | Unknown | PharmGKB |
| REV3L | REV3L | REV3L | FLUOROURACIL | Unknown | PharmGKB |
| REV3L | REV3L | REV3L | CISPLATIN | Unknown | PharmGKB |
| REV3L | REV3L | REV3L | CAPECITABINE | Unknown | PharmGKB |
| KLK3 | KLK3 | KLK3 | ECALLANTIDE | inhibitor | ChemblInteractions |
| KLK3 | KLK3 | KLK3 | APROTININ | inhibitor | ChemblInteractions |
| KLK3 | KLK3 | KLK3 | PROGESTERONE | Unknown | NCI |
| KLK3 | KLK3 | KLK3 | ABARELIX | Unknown | NCI |
| KLK3 | KLK3 | KLK3 | MIFEPRISTONE | Unknown | NCI |
| KLK3 | KLK3 | KLK3 | TAMOXIFEN | Unknown | NCI |
| KLK3 | KLK3 | KLK3 | ETOPOSIDE | Unknown | NCI |
| KLK3 | KLK3 | KLK3 | ANDROSTENEDIONE | Unknown | NCI |
| KLK3 | KLK3 | KLK3 | OCTREOTIDE | Unknown | NCI |
| KLK3 | KLK3 | KLK3 | DOCETAXEL | Unknown | NCI |
| KLK3 | KLK3 | KLK3 | SELENOMETHIONINE | Unknown | NCI |
| KLK3 | KLK3 | KLK3 | VINBLASTINE | Unknown | NCI |
| KLK3 | KLK3 | KLK3 | FLUTAMIDE | Unknown | NCI |
| KLK3 | KLK3 | KLK3 | TOPSALYSIN | Unknown | TdgClinicalTrial\|TTD |
| KLK3 | KLK3 | KLK3 | GENISTEIN | Unknown | NCI |
| KLK3 | KLK3 | KLK3 | BICALUTAMIDE | Unknown | NCI |
| KLK3 | KLK3 | KLK3 | NILUTAMIDE | Unknown | NCI |
| KLK3 | KLK3 | KLK3 | ESTRAMUSTINE | Unknown | NCI |
| CSNK1G2 | CSNK1G2 | CSNK1G2 | CHEMBL225519 | Unknown | DTC |
| CSNK1G2 | CSNK1G2 | CSNK1G2 | TAK-715 | Unknown | DTC |
| CSNK1G2 | CSNK1G2 | CSNK1G2 | SP-600125 | Unknown | DTC |
| CSNK1G2 | CSNK1G2 | CSNK1G2 | ALSTERPAULLONE | Unknown | DTC |
| CSNK1G2 | CSNK1G2 | CSNK1G2 | SB-220025 | Unknown | DTC |
| CSNK1G2 | CSNK1G2 | CSNK1G2 | PF-00562271 | Unknown | DTC |
| CSNK1G2 | CSNK1G2 | CSNK1G2 | SNS-314 | Unknown | DTC |
| CSNK1G2 | CSNK1G2 | CSNK1G2 | LINIFANIB | Unknown | DTC |
| CSNK1G2 | CSNK1G2 | CSNK1G2 | CYC-116 | Unknown | DTC |
| CSNK1G2 | CSNK1G2 | CSNK1G2 | ILORASERTIB | Unknown | DTC |
| CSNK1G2 | CSNK1G2 | CSNK1G2 | (RS)-ROSCOVITINE | Unknown | DTC |
| CSNK1G2 | CSNK1G2 | CSNK1G2 | CENISERTIB | Unknown | DTC |
| PTPN1 | PTPN1 | PTPN1 | OLEANOLIC_ACID | Unknown | DTC |
| PTPN1 | PTPN1 | PTPN1 | ALLYL ISOTHIOCYANATE | Unknown | DTC |
| PTPN1 | PTPN1 | PTPN1 | LICOAGRODIN | Unknown | DTC |
| PTPN1 | PTPN1 | PTPN1 | CHEMBL2207339 | Unknown | DTC |
| PTPN1 | PTPN1 | PTPN1 | TILUDRONIC ACID | Unknown | TdgClinicalTrial\|TEND |
| PTPN1 | PTPN1 | PTPN1 | GLISOFLAVONE | Unknown | DTC |
| PTPN1 | PTPN1 | PTPN1 | INSULIN | Unknown | NCI |
| PTPN1 | PTPN1 | PTPN1 | FLUORESCEIN DIPHOSPHATE | Unknown | DTC |
| PTPN1 | PTPN1 | PTPN1 | LICOAGRONE | Unknown | DTC |
| PTPN1 | PTPN1 | PTPN1 | URSOLIC ACID | Unknown | DTC |
| PTPN1 | PTPN1 | PTPN1 | TRODUSQUEMINE | inhibitor | TdgClinicalTrial\|TTD |
| PTPN1 | PTPN1 | PTPN1 | MOROLIC ACID | Unknown | DTC |
| PTPN1 | PTPN1 | PTPN1 | CHEMBL1162414 | Unknown | DTC |
| PTPN1 | PTPN1 | PTPN1 | LIPIDYL PSEUDOPTERANE A | Unknown | DTC |
| PTPN1 | PTPN1 | PTPN1 | CHEMBL458855 | Unknown | DTC |
| PTPN1 | PTPN1 | PTPN1 | ALDISIN | Unknown | DTC |
| PTPN1 | PTPN1 | PTPN1 | LIPIDYL PSEUDOPTERANE D | Unknown | DTC |
| PTPN1 | PTPN1 | PTPN1 | ANETHOLTRITHION | Unknown | DTC |
| PTPN1 | PTPN1 | PTPN1 | CHEMBL204543 | Unknown | DTC |
| PTPN1 | PTPN1 | PTPN1 | DITHIOLETHIONE | Unknown | DTC |
| PTPN1 | PTPN1 | PTPN1 | ISOBAVACHALCONE | Unknown | DTC |
| PTPN1 | PTPN1 | PTPN1 | OLTIPRAZ | Unknown | DTC |
| PTPN1 | PTPN1 | PTPN1 | MORONIC ACID | Unknown | DTC |
| PTPN1 | PTPN1 | PTPN1 | LOBARIC ACID | Unknown | DTC |
| MGLL | MGLL | MGLL | CHEMBL565711 | Unknown | DTC |
| MGLL | MGLL | MGLL | URB-597 | Unknown | DTC |
| MGLL | MGLL | MGLL | OCTHILINONE | Unknown | DTC |
| MGLL | MGLL | MGLL | CHEMBL76012 | Unknown | DTC |
| F8 | F8 | F8 | TB-402 | inhibitor | TdgClinicalTrial\|ChemblInteractions\|TTD |
| F8 | F8 | F8 | THROMBIN | activator | TdgClinicalTrial |
| F8 | F8 | F8 | EFMOROCTOCOG ALFA | Unknown | TTD |
| F8 | F8 | F8 | DROTRECOGIN ALFA (ACTIVATED) | inhibitor | ChemblInteractions |
| F8 | F8 | F8 | SORBITOL | Unknown | NCI |
| F8 | F8 | F8 | RANITIDINE | Unknown | NCI |
| ATG16L1 | ATG16L1 | ATG16L1 | ADALIMUMAB | Unknown | PharmGKB |
| RHOA | RHOA | RHOA | PRAVASTATIN | Unknown | PharmGKB |
| RHOA | RHOA | RHOA | ROTTLERIN | Unknown | DTC |
| RHOA | RHOA | RHOA | CLAUSINE E | Unknown | DTC |
| RHOA | RHOA | RHOA | SIMVASTATIN | Unknown | PharmGKB |
| IL6R | IL6R | IL6R | TOCILIZUMAB | antibody\|inhibitor | MyCancerGenome\|TdgClinicalTrial\|ChemblInteractions\|TEND\|PharmGKB\|TTD |
| IL6R | IL6R | IL6R | SIRUKUMAB | Unknown | TdgClinicalTrial\|TTD |
| IL6R | IL6R | IL6R | THALIDOMIDE | Unknown | NCI |
| IL6R | IL6R | IL6R | VOBARILIZUMAB | antibody | TTD |
| IL6R | IL6R | IL6R | RALOXIFENE | Unknown | DTC |
| IL6R | IL6R | IL6R | SATRALIZUMAB | antibody\|antagonist | ChemblInteractions\|TTD |
| IL6R | IL6R | IL6R | SARILUMAB | antagonist\|antibody | ChemblInteractions\|TTD |
| IL6R | IL6R | IL6R | BAZEDOXIFENE | Unknown | DTC |
| IL6R | IL6R | IL6R | FLUOROURACIL | Unknown | NCI |
| CACNA1H | CACNA1H | CACNA1H | FLUNARIZINE DIHYDROCHLORIDE | Unknown | DTC |
| CACNA1H | CACNA1H | CACNA1H | ELLIPTECINE | Unknown | DTC |
| CACNA1H | CACNA1H | CACNA1H | CHEMBL592106 | Unknown | DTC |
| CACNA1H | CACNA1H | CACNA1H | NAFTOPIDIL | Unknown | DTC |
| CACNA1H | CACNA1H | CACNA1H | PARAMETHADIONE | blocker | ChemblInteractions |
| CACNA1H | CACNA1H | CACNA1H | PHENSUXIMIDE | blocker | ChemblInteractions |
| CACNA1H | CACNA1H | CACNA1H | CHEMBL374632 | Unknown | DTC |
| CACNA1H | CACNA1H | CACNA1H | CHEMBL1570199 | Unknown | DTC |
| CACNA1H | CACNA1H | CACNA1H | CHEMBL533602 | Unknown | DTC |
| CACNA1H | CACNA1H | CACNA1H | BEPRIDIL HYDROCHLORIDE | blocker | ChemblInteractions |
| CACNA1H | CACNA1H | CACNA1H | FLUNARIZINE | inhibitor | TdgClinicalTrial\|TEND\|TTD |
| CACNA1H | CACNA1H | CACNA1H | MIBEFRADIL | blocker\|inhibitor | TTD |
| CACNA1H | CACNA1H | CACNA1H | GABAPENTIN ENACARBIL | modulator | ChemblInteractions |
| CACNA1H | CACNA1H | CACNA1H | ZONISAMIDE | inhibitor | TdgClinicalTrial |
| CACNA1H | CACNA1H | CACNA1H | ABT-639 | blocker | TTD |
| CACNA1H | CACNA1H | CACNA1H | GABAPENTIN | modulator | ChemblInteractions |
| CACNA1H | CACNA1H | CACNA1H | METHSUXIMIDE | blocker | ChemblInteractions |
| CACNA1H | CACNA1H | CACNA1H | MIBEFRADIL DIHYDROCHLORIDE | blocker | ChemblInteractions |
| CACNA1H | CACNA1H | CACNA1H | CHEMBL526952 | Unknown | DTC |
| CACNA1H | CACNA1H | CACNA1H | ATAGABALIN | modulator | ChemblInteractions |
| CACNA1H | CACNA1H | CACNA1H | OXATOMIDE | Unknown | DTC |
| CACNA1H | CACNA1H | CACNA1H | CHEMBL592566 | Unknown | DTC |
| CACNA1H | CACNA1H | CACNA1H | ETHOSUXIMIDE | blocker | ChemblInteractions |
| CACNA1H | CACNA1H | CACNA1H | PREGABALIN | modulator | ChemblInteractions |
| CACNA1H | CACNA1H | CACNA1H | TRIMETHADIONE | blocker | ChemblInteractions |
| CACNA1H | CACNA1H | CACNA1H | CELECOXIB | Unknown | PharmGKB |
| CACNA1H | CACNA1H | CACNA1H | IMAGABALIN | modulator | ChemblInteractions |
| SH2D1A | SH2D1A | SH2D1A | EMAPALUMAB | Unknown | PharmGKB\|FDA |
| NDUFS4 | NDUFS4 | NDUFS4 | ME-344 | inhibitor | ChemblInteractions |
| NDUFS4 | NDUFS4 | NDUFS4 | NV-128 | inhibitor | ChemblInteractions |
| NDUFS4 | NDUFS4 | NDUFS4 | METFORMIN HYDROCHLORIDE | inhibitor | ChemblInteractions |
| NDUFB1 | NDUFB1 | NDUFB1 | ME-344 | inhibitor | ChemblInteractions |
| NDUFB1 | NDUFB1 | NDUFB1 | NV-128 | inhibitor | ChemblInteractions |
| NDUFB1 | NDUFB1 | NDUFB1 | METFORMIN HYDROCHLORIDE | inhibitor | ChemblInteractions |
| RASA1 | RASA1 | RASA1 | TRAMETINIB | Unknown | CIViC |
| NFE2 | NFE2 | NFE2 | PACLITAXEL | Unknown | NCI |
| ACO1 | ACO1 | ACO1 | ACETYLCYSTEINE | Unknown | NCI |
| DOK5 | DOK5 | DOK5 | DEXAMETHASONE | Unknown | PharmGKB |
| DOK5 | DOK5 | DOK5 | DOXORUBICIN | Unknown | PharmGKB |
| DOK5 | DOK5 | DOK5 | CYTARABINE | Unknown | PharmGKB |
| DOK5 | DOK5 | DOK5 | PEGASPARGASE | Unknown | PharmGKB |
| DOK5 | DOK5 | DOK5 | METHOTREXATE | Unknown | PharmGKB |
| DOK5 | DOK5 | DOK5 | VINCRISTINE | Unknown | PharmGKB |
| DOK5 | DOK5 | DOK5 | DAUNORUBICIN | Unknown | PharmGKB |
| DOK5 | DOK5 | DOK5 | PREDNISONE | Unknown | PharmGKB |
| DOK5 | DOK5 | DOK5 | CYCLOPHOSPHAMIDE | Unknown | PharmGKB |
| DOK5 | DOK5 | DOK5 | THIOGUANINE | Unknown | PharmGKB |
| MGEA5 | OGA | OGA | STREPTOZOCIN | Unknown | NCI |
| NDUFS2 | NDUFS2 | NDUFS2 | NV-128 | inhibitor | ChemblInteractions |
| NDUFS2 | NDUFS2 | NDUFS2 | METFORMIN HYDROCHLORIDE | inhibitor | ChemblInteractions |
| NDUFS2 | NDUFS2 | NDUFS2 | ME-344 | inhibitor | ChemblInteractions |
| CBX1 | CBX1 | CBX1 | BENSERAZIDE | Unknown | DTC |
| CBX1 | CBX1 | CBX1 | ROTENONE | Unknown | DTC |
| CBX1 | CBX1 | CBX1 | NICARDIPINE | Unknown | DTC |
| CBX1 | CBX1 | CBX1 | MITOXANTRONE HYDROCHLORIDE | Unknown | DTC |
| CBX1 | CBX1 | CBX1 | PURPURIN | Unknown | DTC |
| CBX1 | CBX1 | CBX1 | SURAMIN | Unknown | DTC |
| CBX1 | CBX1 | CBX1 | CHEMBL297784 | Unknown | DTC |
| CBX1 | CBX1 | CBX1 | CHEMBL56731 | Unknown | DTC |
| CBX1 | CBX1 | CBX1 | CAPSAZEPINE | Unknown | DTC |
| CBX1 | CBX1 | CBX1 | METITEPINE | Unknown | DTC |
| CBX1 | CBX1 | CBX1 | ATHRAQUINONES A | Unknown | DTC |
| CBX1 | CBX1 | CBX1 | CHEMBL339587 | Unknown | DTC |
| CBX1 | CBX1 | CBX1 | TRIMETHOPRIM | Unknown | DTC |
| CBX1 | CBX1 | CBX1 | DOCEBENONE | Unknown | DTC |
| CBX1 | CBX1 | CBX1 | CHEMBL1567571 | Unknown | DTC |
| CBX1 | CBX1 | CBX1 | DOPAMINE HYDROCHLORIDE | Unknown | DTC |
| CBX1 | CBX1 | CBX1 | CHEMBL1256678 | Unknown | DTC |
| CBX1 | CBX1 | CBX1 | CHEMBL119878 | Unknown | DTC |
| CBX1 | CBX1 | CBX1 | METHYLDOPA (RACEMIC) | Unknown | DTC |
| CBX1 | CBX1 | CBX1 | CHEMBL533602 | Unknown | DTC |
| CBX1 | CBX1 | CBX1 | CHEMBL1451216 | Unknown | DTC |
| CBX1 | CBX1 | CBX1 | GW7647 | Unknown | DTC |
| CBX1 | CBX1 | CBX1 | MIFEPRISTONE | Unknown | DTC |
| CBX1 | CBX1 | CBX1 | EXISULIND | Unknown | DTC |
| CBX1 | CBX1 | CBX1 | OMEPRAZOLE | Unknown | DTC |
| CBX1 | CBX1 | CBX1 | CHEMBL515505 | Unknown | DTC |
| CBX1 | CBX1 | CBX1 | GR-127935 | Unknown | DTC |
| CBX1 | CBX1 | CBX1 | TREQUINSIN | Unknown | DTC |
| CBX1 | CBX1 | CBX1 | CHEMBL578741 | Unknown | DTC |
| CBX1 | CBX1 | CBX1 | ISOXANTHOPTERIN | Unknown | DTC |
| CBX1 | CBX1 | CBX1 | CHEMBL286136 | Unknown | DTC |
| CBX1 | CBX1 | CBX1 | CHEMBL189753 | Unknown | DTC |
| CBX1 | CBX1 | CBX1 | TRIMIPRAMINE | Unknown | DTC |
| CBX1 | CBX1 | CBX1 | CLOFAZIMINE | Unknown | DTC |
| CBX1 | CBX1 | CBX1 | IDARUBICIN | Unknown | DTC |
| CBX1 | CBX1 | CBX1 | FLUPHENAZINE | Unknown | DTC |
| CBX1 | CBX1 | CBX1 | HYDROXYAMINE | Unknown | DTC |
| CBX1 | CBX1 | CBX1 | CLOTRIMAZOLE | Unknown | DTC |
| CBX1 | CBX1 | CBX1 | DISULFIRAM | Unknown | DTC |
| CBX1 | CBX1 | CBX1 | TYRPHOSTIN A9 | Unknown | DTC |
| CBX1 | CBX1 | CBX1 | CHEMBL1627202 | Unknown | DTC |
| CBX1 | CBX1 | CBX1 | PROMETHAZINE | Unknown | DTC |
| CBX1 | CBX1 | CBX1 | RESERPINE | Unknown | DTC |
| CBX1 | CBX1 | CBX1 | PROCHLORPERAZINE | Unknown | DTC |
| CBX1 | CBX1 | CBX1 | CHEMBL1701915 | Unknown | DTC |
| CBX1 | CBX1 | CBX1 | CHEMBL1601846 | Unknown | DTC |
| CBX1 | CBX1 | CBX1 | CHEMBL429095 | Unknown | DTC |
| CBX1 | CBX1 | CBX1 | OXIDOPAMINE HYDROCHLORIDE | Unknown | DTC |
| CBX1 | CBX1 | CBX1 | CHEMBL68534 | Unknown | DTC |
| CBX1 | CBX1 | CBX1 | CHEMBL527584 | Unknown | DTC |
| CBX1 | CBX1 | CBX1 | CHEMBL328710 | Unknown | DTC |
| CBX1 | CBX1 | CBX1 | MYRICETIN | Unknown | DTC |
| CBX1 | CBX1 | CBX1 | RALOXIFENE | Unknown | DTC |
| CBX1 | CBX1 | CBX1 | APOMORPHINE HYDROCHLORIDE HEMIHYDRATE | Unknown | DTC |
| CBX1 | CBX1 | CBX1 | CHEMBL258756 | Unknown | DTC |
| CBX1 | CBX1 | CBX1 | CHEMBL24983 | Unknown | DTC |
| CBX1 | CBX1 | CBX1 | CHEMBL261557 | Unknown | DTC |
| CBX1 | CBX1 | CBX1 | CHEMBL322970 | Unknown | DTC |
| CBX1 | CBX1 | CBX1 | THIORIDAZINE | Unknown | DTC |
| CBX1 | CBX1 | CBX1 | EMODIN | Unknown | DTC |
| CBX1 | CBX1 | CBX1 | CHEMBL505670 | Unknown | DTC |
| CBX1 | CBX1 | CBX1 | ETHOPROPAZINE | Unknown | DTC |
| CBX1 | CBX1 | CBX1 | NORDIHYDROGUAIARETIC ACID | Unknown | DTC |
| CBX1 | CBX1 | CBX1 | DIZOCILPINE | Unknown | DTC |
| CBX1 | CBX1 | CBX1 | PYRONIN Y | Unknown | DTC |
| CBX1 | CBX1 | CBX1 | PAPAVERINE | Unknown | DTC |
| CBX1 | CBX1 | CBX1 | FLUSPIRILENE | Unknown | DTC |
| CBX1 | CBX1 | CBX1 | CHEMBL601364 | Unknown | DTC |
| CBX1 | CBX1 | CBX1 | SB-202190 | Unknown | DTC |
| CBX1 | CBX1 | CBX1 | CHEMBL596380 | Unknown | DTC |
| CBX1 | CBX1 | CBX1 | CHEMBL1989853 | Unknown | DTC |
| CBX1 | CBX1 | CBX1 | CHEMBL1287983 | Unknown | DTC |
| CBX1 | CBX1 | CBX1 | CHEMBL602969 | Unknown | DTC |
| CBX1 | CBX1 | CBX1 | CHEMBL235891 | Unknown | DTC |
| CBX1 | CBX1 | CBX1 | CEFOTAXIME | Unknown | DTC |
| CBX1 | CBX1 | CBX1 | CHEMBL176599 | Unknown | DTC |
| CBX1 | CBX1 | CBX1 | APTIGANEL | Unknown | DTC |
| CBX1 | CBX1 | CBX1 | PYRROLIDINE DITHIOCARBAMATE | Unknown | DTC |
| CBX1 | CBX1 | CBX1 | CHEMBL1408276 | Unknown | DTC |
| CBX1 | CBX1 | CBX1 | CHEMBL463783 | Unknown | DTC |
| CBX1 | CBX1 | CBX1 | LEVODOPA | Unknown | DTC |
| CBX1 | CBX1 | CBX1 | CHEMBL88553 | Unknown | DTC |
| CBX1 | CBX1 | CBX1 | SARCOLYSIN | Unknown | DTC |
| CBX1 | CBX1 | CBX1 | INDIRUBIN-3-MONOXIME | Unknown | DTC |
| CBX1 | CBX1 | CBX1 | RABEPRAZOLE SODIUM | Unknown | DTC |
| CBX1 | CBX1 | CBX1 | TETRYDAMINE | Unknown | DTC |
| CBX1 | CBX1 | CBX1 | CHEMBL179611 | Unknown | DTC |
| CBX1 | CBX1 | CBX1 | PYROGALLOL RED | Unknown | DTC |
| CBX1 | CBX1 | CBX1 | PIMOZIDE | Unknown | DTC |
| CBX1 | CBX1 | CBX1 | FLUPENTIXOL | Unknown | DTC |
| CBX1 | CBX1 | CBX1 | CHEMBL493863 | Unknown | DTC |
| CBX1 | CBX1 | CBX1 | CHEMBL26320 | Unknown | DTC |
| CBX1 | CBX1 | CBX1 | R-N-PROPYLNORAPOMORPHINE | Unknown | DTC |
| CBX1 | CBX1 | CBX1 | MORIN | Unknown | DTC |
| CBX1 | CBX1 | CBX1 | CHEMBL275177 | Unknown | DTC |
| CBX1 | CBX1 | CBX1 | CHEMBL1287980 | Unknown | DTC |
| CBX1 | CBX1 | CBX1 | CLOZAPINE | Unknown | DTC |
| CBX1 | CBX1 | CBX1 | CHEMBL403183 | Unknown | DTC |
| CBX1 | CBX1 | CBX1 | TETRADECYLTHIOACETIC ACID | Unknown | DTC |
| CBX1 | CBX1 | CBX1 | OXOLINIC ACID | Unknown | DTC |
| CBX1 | CBX1 | CBX1 | DEPHOSTATIN | Unknown | DTC |
| CBX1 | CBX1 | CBX1 | FLUPIRTINE | Unknown | DTC |
| CBX1 | CBX1 | CBX1 | SENNOSIDE B | Unknown | DTC |
| CBX1 | CBX1 | CBX1 | CHEMBL1256813 | Unknown | DTC |
| CBX1 | CBX1 | CBX1 | CHEMBL538542 | Unknown | DTC |
| CBX1 | CBX1 | CBX1 | FENRETINIDE | Unknown | DTC |
| CBX1 | CBX1 | CBX1 | OXYTETRACYCLINE | Unknown | DTC |
| CBX1 | CBX1 | CBX1 | NIFEDIPINE | Unknown | DTC |
| CBX1 | CBX1 | CBX1 | ISOPROTERENOL | Unknown | DTC |
| CBX1 | CBX1 | CBX1 | CHEMBL53898 | Unknown | DTC |
| CBX1 | CBX1 | CBX1 | FIDUXOSIN | Unknown | DTC |
| CBX1 | CBX1 | CBX1 | EBSELEN | Unknown | DTC |
| CBX1 | CBX1 | CBX1 | METHYLDOPA | Unknown | DTC |
| CBX1 | CBX1 | CBX1 | TYRPHOSTIN 51 | Unknown | DTC |
| CBX1 | CBX1 | CBX1 | CHEMBL312032 | Unknown | DTC |
| CBX1 | CBX1 | CBX1 | VANOXERINE | Unknown | DTC |
| CBX1 | CBX1 | CBX1 | ROTTLERIN | Unknown | DTC |
| CBX1 | CBX1 | CBX1 | GOSSYPOL | Unknown | DTC |
| CBX1 | CBX1 | CBX1 | ARACHIDONOYL GLYCINE | Unknown | DTC |
| CBX1 | CBX1 | CBX1 | CHEMBL1079460 | Unknown | DTC |
| CBX1 | CBX1 | CBX1 | TRIFLUOPERAZINE | Unknown | DTC |
| CBX1 | CBX1 | CBX1 | PHENOXYBENZAMINE | Unknown | DTC |
| CBX1 | CBX1 | CBX1 | CHEMBL523200 | Unknown | DTC |
| CBX1 | CBX1 | CBX1 | LERCANIDIPINE | Unknown | DTC |
| CBX1 | CBX1 | CBX1 | CHEMBL1519374 | Unknown | DTC |
| CBX1 | CBX1 | CBX1 | PD-169316 | Unknown | DTC |
| CBX1 | CBX1 | CBX1 | CHEMBL606166 | Unknown | DTC |
| CBX1 | CBX1 | CBX1 | CHEMBL1270169 | Unknown | DTC |
| CBX1 | CBX1 | CBX1 | CHEMBL56393 | Unknown | DTC |
| CBX1 | CBX1 | CBX1 | WIN-552122 | Unknown | DTC |
| CBX1 | CBX1 | CBX1 | AMBUNOL | Unknown | DTC |
| CBX1 | CBX1 | CBX1 | TAMOXIFEN | Unknown | DTC |
| CBX1 | CBX1 | CBX1 | RUFINAMIDE | Unknown | DTC |
| CBX1 | CBX1 | CBX1 | SB-242084 | Unknown | DTC |
| CBX1 | CBX1 | CBX1 | NAFTOPIDIL | Unknown | DTC |
| CBX1 | CBX1 | CBX1 | TAXIFOLIN | Unknown | DTC |
| CBX1 | CBX1 | CBX1 | CALCIMYCIN | Unknown | DTC |
| CBX1 | CBX1 | CBX1 | CLOROTEPINE | Unknown | DTC |
| CBX1 | CBX1 | CBX1 | CHEMBL2095095 | Unknown | DTC |
| CBX1 | CBX1 | CBX1 | CHEMBL72365 | Unknown | DTC |
| CBX1 | CBX1 | CBX1 | PERPHENAZINE | Unknown | DTC |
| CBX1 | CBX1 | CBX1 | CANTHARIDIC_ACID | Unknown | DTC |
| CBX1 | CBX1 | CBX1 | CHEMBL1310138 | Unknown | DTC |
| CBX1 | CBX1 | CBX1 | DIAMIDE | Unknown | DTC |
| CBX1 | CBX1 | CBX1 | GUAIAZULEN | Unknown | DTC |
| CBX1 | CBX1 | CBX1 | 4-CHLOROMERCURIBENZOIC ACID | Unknown | DTC |
| CBX1 | CBX1 | CBX1 | CHEMBL67535 | Unknown | DTC |
| CBX1 | CBX1 | CBX1 | CHEMBL576409 | Unknown | DTC |
| CBX1 | CBX1 | CBX1 | CARISOPRODOL | Unknown | DTC |
| CBX1 | CBX1 | CBX1 | TRANSTORINE | Unknown | DTC |
| CBX1 | CBX1 | CBX1 | AMSACRINE | Unknown | DTC |
| CBX1 | CBX1 | CBX1 | CHEMBL472940 | Unknown | DTC |
| CBX1 | CBX1 | CBX1 | MITOXANTRONE | Unknown | DTC |
| CBX1 | CBX1 | CBX1 | GUANIDINONALTRINDOLE | Unknown | DTC |
| CBX1 | CBX1 | CBX1 | CHEMBL93655 | Unknown | DTC |
| CBX1 | CBX1 | CBX1 | CHEMBL547483 | Unknown | DTC |
| CBX1 | CBX1 | CBX1 | CHEMBL586000 | Unknown | DTC |
| CBX1 | CBX1 | CBX1 | METERGOLINE | Unknown | DTC |
| CBX1 | CBX1 | CBX1 | CYCLOPHOSPHAMIDE | Unknown | DTC |
| CBX1 | CBX1 | CBX1 | LY-294002 | Unknown | DTC |
| CBX1 | CBX1 | CBX1 | PYRIDOSTIGMINE | Unknown | DTC |
| CBX1 | CBX1 | CBX1 | ELLIPTECINE | Unknown | DTC |
| CBX1 | CBX1 | CBX1 | CHEMBL64239 | Unknown | DTC |
| CBX1 | CBX1 | CBX1 | N6-PHENYLADENOSINE | Unknown | DTC |
| CBX1 | CBX1 | CBX1 | TYRPHOSTIN AG 879 | Unknown | DTC |
| CBX1 | CBX1 | CBX1 | AMIODARONE | Unknown | DTC |
| CBX1 | CBX1 | CBX1 | CYSTAMINE | Unknown | DTC |
| CBX1 | CBX1 | CBX1 | AURINTRICARBOXYLIC ACID | Unknown | DTC |
| CBX1 | CBX1 | CBX1 | FENOLDOPAM | Unknown | DTC |
| CBX1 | CBX1 | CBX1 | CISPLATIN | Unknown | DTC |
| CBX1 | CBX1 | CBX1 | RITANSERIN | Unknown | DTC |
| CBX1 | CBX1 | CBX1 | CHEMBL258767 | Unknown | DTC |
| CBX1 | CBX1 | CBX1 | CHEMBL73711 | Unknown | DTC |
| CBX1 | CBX1 | CBX1 | KETOCONAZOLE | Unknown | DTC |
| CBX1 | CBX1 | CBX1 | HOMOVANILLIC ACID | Unknown | DTC |
| CBX1 | CBX1 | CBX1 | BAY-11-7085 | Unknown | DTC |
| CBX1 | CBX1 | CBX1 | CHEMBL56543 | Unknown | DTC |
| CBX1 | CBX1 | CBX1 | CHEMBL1486366 | Unknown | DTC |
| CBX1 | CBX1 | CBX1 | CHEMBL1412489 | Unknown | DTC |
| CBX1 | CBX1 | CBX1 | CYSTAMINE HYDROCHLORIDE | Unknown | DTC |
| CBX1 | CBX1 | CBX1 | CHEMBL588038 | Unknown | DTC |
| CBX1 | CBX1 | CBX1 | CAMBINOL | Unknown | DTC |
| CBX1 | CBX1 | CBX1 | CHEMBL175266 | Unknown | DTC |
| CBX1 | CBX1 | CBX1 | CHEMBL590186 | Unknown | DTC |
| CBX1 | CBX1 | CBX1 | NAPROXEN | Unknown | DTC |
| CBX1 | CBX1 | CBX1 | E-64 | Unknown | DTC |
| CBX1 | CBX1 | CBX1 | PICEATANNOL | Unknown | DTC |
| CBX1 | CBX1 | CBX1 | CHEMBL580155 | Unknown | DTC |
| PCYT1A | PCYT1A | PCYT1A | LAMIVUDINE | Unknown | PharmGKB |
| SOD2 | SOD2 | SOD2 | CYCLOPHOSPHAMIDE | Unknown | PharmGKB |
| SOD2 | SOD2 | SOD2 | ASPARAGINASE | Unknown | PharmGKB |
| SOD2 | SOD2 | SOD2 | DIACETYLMORPHINE | Unknown | PharmGKB |
| SOD2 | SOD2 | SOD2 | PACLITAXEL | Unknown | PharmGKB |
| SOD2 | SOD2 | SOD2 | METHOTREXATE | Unknown | PharmGKB |
| FBXL17 | FBXL17 | FBXL17 | HYDROCHLOROTHIAZIDE | Unknown | PharmGKB |
| SLCO2B1 | SLCO2B1 | SLCO2B1 | ROSUVASTATIN | Unknown | PharmGKB |
| SLCO2B1 | SLCO2B1 | SLCO2B1 | ISOSILYBIN B | Unknown | DTC |
| SLCO2B1 | SLCO2B1 | SLCO2B1 | ELTROMBOPAG | Unknown | DTC |
| SLCO2B1 | SLCO2B1 | SLCO2B1 | SILIBININ | Unknown | DTC |
| SLCO2B1 | SLCO2B1 | SLCO2B1 | FLUVASTATIN | Unknown | PharmGKB |
| SLCO2B1 | SLCO2B1 | SLCO2B1 | SULFOBROMOPHTHALEIN | Unknown | DTC |
| SLCO2B1 | SLCO2B1 | SLCO2B1 | ISOSILYBIN A | Unknown | DTC |
| SLCO2B1 | SLCO2B1 | SLCO2B1 | SCUTELLARIN | Unknown | DTC |
| SLCO2B1 | SLCO2B1 | SLCO2B1 | PRAVASTATIN | Unknown | PharmGKB |
| SLCO2B1 | SLCO2B1 | SLCO2B1 | ROSIGLITAZONE | Unknown | NCI |
| SLCO2B1 | SLCO2B1 | SLCO2B1 | ATORVASTATIN | Unknown | PharmGKB |
| SLCO2B1 | SLCO2B1 | SLCO2B1 | SILICRISTIN | Unknown | DTC |
| SLCO2B1 | SLCO2B1 | SLCO2B1 | FEXOFENADINE | Unknown | PharmGKB |
| SLCO2B1 | SLCO2B1 | SLCO2B1 | SIMVASTATIN | Unknown | PharmGKB |
| SLCO2B1 | SLCO2B1 | SLCO2B1 | SILYBIN B | Unknown | DTC |
| SLCO2B1 | SLCO2B1 | SLCO2B1 | MONTELUKAST | Unknown | PharmGKB |
| SLCO2B1 | SLCO2B1 | SLCO2B1 | LOVASTATIN | Unknown | PharmGKB |
| SLCO2B1 | SLCO2B1 | SLCO2B1 | SILYDIANIN | Unknown | DTC |
| SCN5A | SCN5A | SCN5A | ETHOTOIN | blocker\|inhibitor | TdgClinicalTrial\|ChemblInteractions\|TEND\|TTD |
| SCN5A | SCN5A | SCN5A | DRONEDARONE HYDROCHLORIDE | blocker | ChemblInteractions |
| SCN5A | SCN5A | SCN5A | APRINDINE | inhibitor | TdgClinicalTrial |
| SCN5A | SCN5A | SCN5A | FOSPHENYTOIN | inhibitor | TdgClinicalTrial\|TEND\|TTD |
| SCN5A | SCN5A | SCN5A | SAFINAMIDE | Unknown | TdgClinicalTrial |
| SCN5A | SCN5A | SCN5A | PROPAFENONE HYDROCHLORIDE | blocker | ChemblInteractions |
| SCN5A | SCN5A | SCN5A | FLECAINIDE | inhibitor | TdgClinicalTrial\|TEND\|PharmGKB |
| SCN5A | SCN5A | SCN5A | ENCAINIDE | inhibitor | TdgClinicalTrial\|TEND |
| SCN5A | SCN5A | SCN5A | CARBAMAZEPINE | inhibitor\|blocker | TdgClinicalTrial\|ChemblInteractions\|TEND |
| SCN5A | SCN5A | SCN5A | OXCARBAZEPINE | inhibitor\|blocker | TdgClinicalTrial\|ChemblInteractions\|TEND |
| SCN5A | SCN5A | SCN5A | LIDOCAINE | blocker\|inhibitor | TdgClinicalTrial\|ChemblInteractions\|TEND\|PharmGKB |
| SCN5A | SCN5A | SCN5A | DIBUCAINE | inhibitor | TdgClinicalTrial\|TEND\|TTD |
| SCN5A | SCN5A | SCN5A | PHENACEMIDE | blocker | ChemblInteractions |
| SCN5A | SCN5A | SCN5A | DISOPYRAMIDE | inhibitor | TdgClinicalTrial\|TEND\|PharmGKB\|TTD |
| SCN5A | SCN5A | SCN5A | COCAINE | inhibitor | TdgClinicalTrial\|TEND\|PharmGKB |
| SCN5A | SCN5A | SCN5A | PROPARACAINE HYDROCHLORIDE | blocker | ChemblInteractions |
| SCN5A | SCN5A | SCN5A | PROCAINAMIDE | inhibitor | TdgClinicalTrial\|TEND\|PharmGKB |
| SCN5A | SCN5A | SCN5A | MEPHENYTOIN | blocker\|inhibitor | TdgClinicalTrial\|ChemblInteractions\|TEND\|TTD |
| SCN5A | SCN5A | SCN5A | MEXILETINE | inhibitor | TdgClinicalTrial\|TEND\|PharmGKB\|TTD |
| SCN5A | SCN5A | SCN5A | PRILOCAINE | inhibitor\|blocker | TdgClinicalTrial\|ChemblInteractions\|TEND\|TTD |
| SCN5A | SCN5A | SCN5A | FOSPHENYTOIN SODIUM | blocker | ChemblInteractions |
| SCN5A | SCN5A | SCN5A | BENZONATATE | antagonist | TdgClinicalTrial\|TEND\|TTD |
| SCN5A | SCN5A | SCN5A | BEPRIDIL | Unknown | TdgClinicalTrial\|TEND |
| SCN5A | SCN5A | SCN5A | MORICIZINE | inhibitor | TdgClinicalTrial\|TEND\|TTD |
| SCN5A | SCN5A | SCN5A | QUINIDINE POLYGALACTURONATE | blocker | ChemblInteractions |
| SCN5A | SCN5A | SCN5A | DYCLONINE HYDROCHLORIDE | blocker | ChemblInteractions |
| SCN5A | SCN5A | SCN5A | RILUZOLE | blocker\|inhibitor | TdgClinicalTrial\|ChemblInteractions |
| SCN5A | SCN5A | SCN5A | INDECAINIDE | inhibitor | TdgClinicalTrial\|TEND\|TTD |
| SCN5A | SCN5A | SCN5A | LITHIUM |  | PharmGKB |
| SCN5A | SCN5A | SCN5A | ERLOSAMIDE | blocker | ChemblInteractions |
| SCN5A | SCN5A | SCN5A | PHENAZOPYRIDINE HYDROCHLORIDE | blocker | ChemblInteractions |
| SCN5A | SCN5A | SCN5A | ARTICAINE HYDROCHLORIDE | blocker | ChemblInteractions |
| SCN5A | SCN5A | SCN5A | ETIDOCAINE HYDROCHLORIDE | blocker | ChemblInteractions |
| SCN5A | SCN5A | SCN5A | MORICIZINE HYDROCHLORIDE | blocker | ChemblInteractions |
| SCN5A | SCN5A | SCN5A | RUFINAMIDE | blocker | ChemblInteractions |
| SCN5A | SCN5A | SCN5A | PHENYTOIN SODIUM | blocker | ChemblInteractions |
| SCN5A | SCN5A | SCN5A | NERISPIRDINE | blocker | ChemblInteractions |
| SCN5A | SCN5A | SCN5A | CLOMIPRAMINE | Unknown | DTC |
| SCN5A | SCN5A | SCN5A | HEXYLCAINE HYDROCHLORIDE | blocker | ChemblInteractions |
| SCN5A | SCN5A | SCN5A | QUINIDINE | inhibitor | TdgClinicalTrial\|TEND\|PharmGKB |
| SCN5A | SCN5A | SCN5A | PROPAFENONE | inhibitor | TdgClinicalTrial\|TEND\|PharmGKB |
| SCN5A | SCN5A | SCN5A | PRILOCAINE HYDROCHLORIDE | blocker | ChemblInteractions |
| SCN5A | SCN5A | SCN5A | RALFINAMIDE | blocker | ChemblInteractions |
| SCN5A | SCN5A | SCN5A | DYCLONINE | Unknown | TTD |
| SCN5A | SCN5A | SCN5A | EVENAMIDE | blocker | ChemblInteractions |
| SCN5A | SCN5A | SCN5A | QUINIDINE GLUCONATE | blocker | ChemblInteractions |
| SCN5A | SCN5A | SCN5A | LAMOTRIGINE | blocker | ChemblInteractions |
| SCN5A | SCN5A | SCN5A | PHENYTOIN | blocker\|inhibitor | TdgClinicalTrial\|ChemblInteractions\|TEND |
| SCN5A | SCN5A | SCN5A | PRIMIDONE | blocker | ChemblInteractions |
| SCN5A | SCN5A | SCN5A | DISOPYRAMIDE PHOSPHATE | blocker | ChemblInteractions |
| SCN5A | SCN5A | SCN5A | PILSICAINIDE | Unknown | PharmGKB |
| SCN5A | SCN5A | SCN5A | LOMERIZINE | Unknown | TTD |
| SCN5A | SCN5A | SCN5A | BENOXINATE HYDROCHLORIDE | blocker | ChemblInteractions |
| SCN5A | SCN5A | SCN5A | TOPIRAMATE | blocker | ChemblInteractions |
| SCN5A | SCN5A | SCN5A | RANOLAZINE | blocker\|inhibitor | TdgClinicalTrial\|ChemblInteractions\|TEND |
| SCN5A | SCN5A | SCN5A | IRAMPANEL | blocker | ChemblInteractions |
| SCN5A | SCN5A | SCN5A | LIDOCAINE HYDROCHLORIDE | blocker | ChemblInteractions |
| SCN5A | SCN5A | SCN5A | HEXYLCAINE | inhibitor | TdgClinicalTrial\|TEND\|TTD |
| SCN5A | SCN5A | SCN5A | MEPIVACAINE HYDROCHLORIDE | blocker | ChemblInteractions |
| SCN5A | SCN5A | SCN5A | MERETHOXYLLINE PROCAINE | blocker | ChemblInteractions |
| SCN5A | SCN5A | SCN5A | TETRODOTOXIN | Unknown | TdgClinicalTrial\|TTD |
| SCN5A | SCN5A | SCN5A | TETRACAINE | blocker | ChemblInteractions |
| SCN5A | SCN5A | SCN5A | ZONISAMIDE | blocker\|inhibitor | TdgClinicalTrial\|ChemblInteractions |
| SCN5A | SCN5A | SCN5A | PROPOXYCAINE HYDROCHLORIDE | blocker | ChemblInteractions |
| SCN5A | SCN5A | SCN5A | ESLICARBAZEPINE | blocker | TdgClinicalTrial\|ChemblInteractions |
| SCN5A | SCN5A | SCN5A | ESLICARBAZEPINE ACETATE | blocker | ChemblInteractions |
| SCN5A | SCN5A | SCN5A | DIBUCAINE HYDROCHLORIDE | blocker | ChemblInteractions |
| SCN5A | SCN5A | SCN5A | TOCAINIDE | inhibitor | TdgClinicalTrial\|TEND |
| SCN5A | SCN5A | SCN5A | AZD1305 | blocker | ChemblInteractions |
| SCN5A | SCN5A | SCN5A | ORPHENADRINE CITRATE | blocker | ChemblInteractions |
| SCN5A | SCN5A | SCN5A | PROCAINAMIDE HYDROCHLORIDE | blocker | ChemblInteractions |
| SCN5A | SCN5A | SCN5A | NKTR-171 | blocker | ChemblInteractions |
| SCN5A | SCN5A | SCN5A | AMIODARONE | Unknown | PharmGKB |
| SCN5A | SCN5A | SCN5A | TOCAINIDE HYDROCHLORIDE | blocker | ChemblInteractions |
| SCN5A | SCN5A | SCN5A | QUINIDINE SULFATE | blocker | ChemblInteractions |
| SCN5A | SCN5A | SCN5A | AZD7009 | blocker | ChemblInteractions |
| SCN5A | SCN5A | SCN5A | FLECAINIDE ACETATE | blocker | ChemblInteractions |
| SCN5A | SCN5A | SCN5A | LICARBAZEPINE | Unknown | TdgClinicalTrial |
| SCN5A | SCN5A | SCN5A | PROCAINE HYDROCHLORIDE | blocker | ChemblInteractions |
| SCN5A | SCN5A | SCN5A | ROPIVACAINE HYDROCHLORIDE | blocker | ChemblInteractions |
| SCN5A | SCN5A | SCN5A | INDECAINIDE HYDROCHLORIDE | blocker | ChemblInteractions |
| SCN5A | SCN5A | SCN5A | CHLOROPROCAINE HYDROCHLORIDE | blocker | ChemblInteractions |
| SCN5A | SCN5A | SCN5A | ORPHENADRINE HYDROCHLORIDE | blocker | ChemblInteractions |
